# State-of-the-art photodynamic therapy for malignant gliomas: innovations in photosensitizers and combined therapeutic approaches

**DOI:** 10.37349/etat.2025.1002303

**Published:** 2025-03-28

**Authors:** Bruno A. Cesca, Kali Pellicer San Martin, Matías D. Caverzan, Paula M. Oliveda, Luis E. Ibarra

**Affiliations:** IRCCS Istituto Romagnolo per lo Studio dei Tumori (IRST) “Dino Amadori”, Italy; ^1^Departamento de Biología Molecular, Facultad de Ciencias Exactas, Fisicoquímicas y Naturales, Universidad Nacional de Río Cuarto (UNRC), Rio Cuarto X5800BIA, Argentina; ^2^Instituto de Investigaciones en Tecnologías Energéticas y Materiales Avanzados (IITEMA), Universidad Nacional de Río Cuarto (UNRC) y Consejo Nacional de Investigaciones Científicas y Técnicas (CONICET), Río Cuarto X5800BIA, Argentina; ^3^Departamento de Patología Animal, Facultad de Agronomía y Veterinaria, Universidad Nacional de Río Cuarto (UNRC), Rio Cuarto X5800BIA, Argentina; ^4^Instituto de Biotecnología Ambiental y Salud (INBIAS), Universidad Nacional de Río Cuarto (UNRC) y Consejo Nacional de Investigaciones Científicas y Técnicas (CONICET), Rio Cuarto X5800BIA, Argentina

**Keywords:** Glioblastoma, nanoparticles, photosensitizers, resistance mechanisms, combination therapy

## Abstract

Glioblastoma (GBM), the most aggressive and lethal primary brain tumor, poses a significant therapeutic challenge due to its highly invasive nature and resistance to conventional therapies, including surgery, chemotherapy, and radiotherapy. Despite advances in standard treatments, patient survival remains limited, requiring the exploration of innovative strategies. Photodynamic therapy (PDT) has emerged as a promising approach, leveraging light-sensitive photosensitizers (PSs), molecular oxygen, and specific light wavelengths to generate reactive oxygen species (ROS) that selectively induce tumor cell death. Originally developed for skin cancer, PDT has evolved to target more complex malignancies, including GBM. The refinement of second- and third-generation PS, coupled with advancements in nanotechnology, has significantly improved PDT’s selectivity, bioavailability, and therapeutic efficacy. Moreover, the combination of PDT with chemotherapy, targeted therapy, and immunotherapy, among other therapeutic modalities, has shown potential in enhancing therapeutic outcomes. This review provides a comprehensive analysis of the preclinical and clinical applications of PDT in GBM, detailing its mechanisms of action, the evolution of PS, and novel combinatory strategies that optimize treatment efficacy. However, several challenges remain, including overcoming GBM-associated hypoxia, enhancing PS delivery across the blood-brain barrier, and mitigating tumor resistance mechanisms. The integration of PDT with molecular and genetic insight, alongside cutting-edge nanotechnology-based delivery systems, may revolutionize GBM treatment, offering new prospects for improved patient survival and quality of life.

## Graphical abstract

**Graphical abstract.**
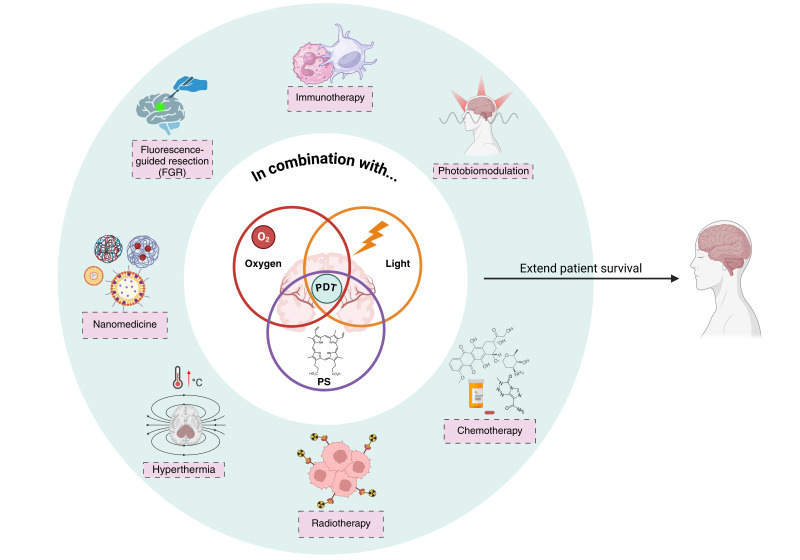
** Integrating PDT with conventional and emerging therapies.** PDT: photodynamic therapy; PS: photosensitizer. Created in BioRender. Ibarra, L. (2025) https://BioRender.com/j94m405

## Introduction

It is widely recognized in the scientific community that treating glioblastoma (GBM) presents significant challenges, where the therapeutic regimen known as the Stupp protocol only results in a survival of 14–16 months [1]. This substantial issue gives rise to numerous research opportunities in the search for alternative therapies for this condition [2]. GBM is the most aggressive and lethal form of primary brain tumor, with a median survival of less than 15 months despite current standard treatments involving surgery, radiotherapy (RT), and chemotherapy (CTX). Recent advances in molecular and genetic profiling have improved our understanding of GBM’s complex biology, highlighting the role of mutations in the isocitrate dehydrogenase 1 and 2 genes (*IDH1/2*), *MGMT* promoter methylation, and other markers that are central to GBM’s pathogenesis and resistance mechanisms [3]. However, effective treatments that can significantly extend patient survival remain limited. One promising avenue is photodynamic therapy (PDT), which is a minimally invasive medical treatment that leverages the interaction between a photosensitizing agent, light, and molecular oxygen (O_2_) to produce reactive O_2_ species (ROS) capable of selectively destroying targeted cells [4]. The therapeutic potential of PDT was first recognized over a century ago, but its clinical application began to gain significant momentum in the 1970s with the discovery of porphyrins as effective photosensitizers (PSs). Initially developed for the treatment of skin cancers, PDT has evolved over the decades to include a broader range of applications, from non-oncological conditions to various types of cancer, including GBM, one of the most aggressive brain tumors [5, 6]. Early generations of PSs, like hematoporphyrin (HP) derivatives (HpD), laid the foundation for PDT by demonstrating the ability to accumulate in malignant tissues. However, their limited tissue penetration and prolonged photosensitivity hindered widespread use. As research progressed, second-generation PSs such as 5-aminolevulinic acid (5-ALA) and its derivatives improved selectivity and efficiency, allowing deeper tissue targeting and fewer side effects. Today, third-generation PSs are being developed, often in conjunction with nanotechnology, to further enhance targeting capabilities, improve bioavailability, and minimize dark toxicity but in the best scenario, there remain in preclinical trials [7]. Nanotechnology offers significant advantages in the development of PSs for PDT, enabling enhanced precision, efficiency, and safety in treatment [8]. By encapsulating PSs within nanocarriers, their solubility, stability, and bioavailability can be greatly improved, overcoming limitations associated with traditional PSs from the first generations, such as poor water solubility and rapid degradation. Nanoparticles (NPs) also provide the opportunity for image processing [9], diagnosis [10], treatment [11], targeted delivery [12], reducing off-target effects [13], gene transferring [14], and minimizing damage to healthy tissues [15], particularly in sensitive areas like the brain. Cancer therapy targeting tactics can be classified into passive and active approaches, each possessing distinct advantages and limitations in the context of brain tumors. Passive targeting utilizes the enhanced permeability and retention (EPR) phenomenon, whereby NPs aggregate in tumor tissues owing to compromised vasculature and deficient lymphatic outflow. This method encounters obstacles in brain tumors because of the blood-brain barrier (BBB) and the diverse characteristics of tumor vasculature, which frequently restrict efficient NP delivery. Active targeting employs ligands or antibodies coupled to NPs to selectively attach to overexpressed receptors on tumor cells and endothelial cells of the BBB, thereby promoting receptor-mediated transcytosis [7]. Although active targeting provides enhanced selectivity and efficiency, it is frequently impeded by inadequate penetration into the tumor bulk and possible off-target effects. Integrating both tactics with modern delivery systems and BBB-disrupting approaches, such as targeted ultrasound (US) or receptor-mediated transcytosis, offers potential to overcome these constraints. These methodologies can enhance the accuracy and therapeutic efficacy of NP-based interventions for brain tumors, promoting the advancement of more effective treatments.

Furthermore, nanotechnology allows for multifunctional systems, integrating imaging agents, targeting ligands, and therapeutic molecules into a single platform, thus enabling theranostic applications. These advancements not only enhance the therapeutic efficacy of PDT but also allow for more precise control over treatment parameters, such as light activation and ROS generation, paving the way for more personalized and effective cancer therapies.

Over the decades, the clinical applications of PDT have expanded, with approvals for treating various cancers, including lung, esophageal, and skin cancers, as well as ongoing trials for its use in treating brain tumors like GBM. However, many of the PSs tested in clinical studies for glioma patients belong to the first and second generations. Some of these studies show moderate efficacy and increased patient survival, but many other studies have not been completed, or the results remain inconclusive.

The growing understanding of tumor biology and advances in molecular techniques have enabled researchers to fine-tune PDT protocols, making it a promising complementary approach in cancer therapy, especially for tumors resistant to conventional treatments. This review explores the potential of PDT in GBM treatment with the most recent developments, focusing on its mechanisms, challenges, and the evolving landscape of PSs across different generations. For the writing of this review, a comprehensive analysis of articles related to the topic was conducted through a systematic search of scientific articles, reviews, preclinical studies, and clinical trials. The main databases consulted were PubMed, Scopus, Web of Science, and Google Scholar. Most of the included articles were published within the last 5–10 years, with a focus on research detailing the combined use of PDT with other therapies. The keywords used in the search were: malignant gliomas, GBM, photoassisted therapy, PDT, synergism, and pro-oxidant therapies, in all possible combinations. Additionally, a special focus was placed on the combinatory option with other therapies that have been investigated in recent years, demonstrating the versatility of light-assisted technology in damaging tumor cells.

## Therapeutic implications of malignant glioma characteristics

Tumors located in the central nervous system (CNS) affect both the brain and spinal cord, with GBM being among the most aggressive and incurable of these malignancies [16]. GBM are highly vascular tumors characterized histologically by necrosis areas, microvascular proliferation, high mitotic rates, invasiveness, pleomorphism, and nuclear atypia [17–19]. GBM is generally considered a spontaneous tumor. The only confirmed risk factor at present is exposure to high-dose ionizing radiation [20]. Certain hereditary syndromes, including neurofibromatosis, Li-Fraumeni syndrome, and von Hippel-Lindau syndrome, have been shown to correlate with a minor percentage of GBM patients [21, 22]. Its development was associated with dysregulation of the G1/S checkpoint in the cell cycle. Additionally, disruptions in tumor suppressor pathways, including p53 and retinoblastoma (Rb), as well as mutations in genes regulating Rb, play a crucial role in the pathogenesis of these tumors [23]. There is a correlation between human cytomegalovirus (HCMV) and GBM development, as HCMV-encoded proteins activate intracellular signaling pathways involved in mitogenesis, mutagenesis, apoptosis, inflammation, and angiogenesis [21].

Malignant gliomas have long been categorized into two categories based on variations in molecular markers, clinical presentations, and disease progression. Primary GBM, commonly referred to as de novo GBM, develops without any apparent precursor lesions [24]. These are more common, typically diagnosed in patients over 50 years of age, and are associated with a more aggressive clinical course. These tumors have a unique molecular profile characterized by specific genetic alterations, such as mutations in the *TERT* promoter gene, loss of chromosome 10, suppression of the *PTEN* gene, amplification of the *EGFR* gene, and the presence of the *EGFRvIII* mutation. These genetic alterations promote tumor growth and activate the Shc-Grb2-Ras and phosphatidylinositol 3-kinase (PI3K) signaling pathways, resulting in increased ability to form tumors, cell division, and resistance to cell death. It is important to note that primary GBM tumors do not have mutations in the genes *IDH1/2* [25]. In contrast, secondary GBM develops from pre-existing low-grade or anaplastic astrocytomas and generally has a more favorable clinical course. These tumors are characterized by mutations in codon 132 of *IDH1* or codon 172 of *IDH2* (*IDH1/2* mutant), which inhibit the activity of α-KG-dependent dioxygenases—key enzymes involved in hypoxia detection, histone deacetylation, and DNA methylation. Additionally, secondary GBM frequently exhibits mutations in the *ATRX* and *TP53* genes [23, 25].

**Figure 1:**
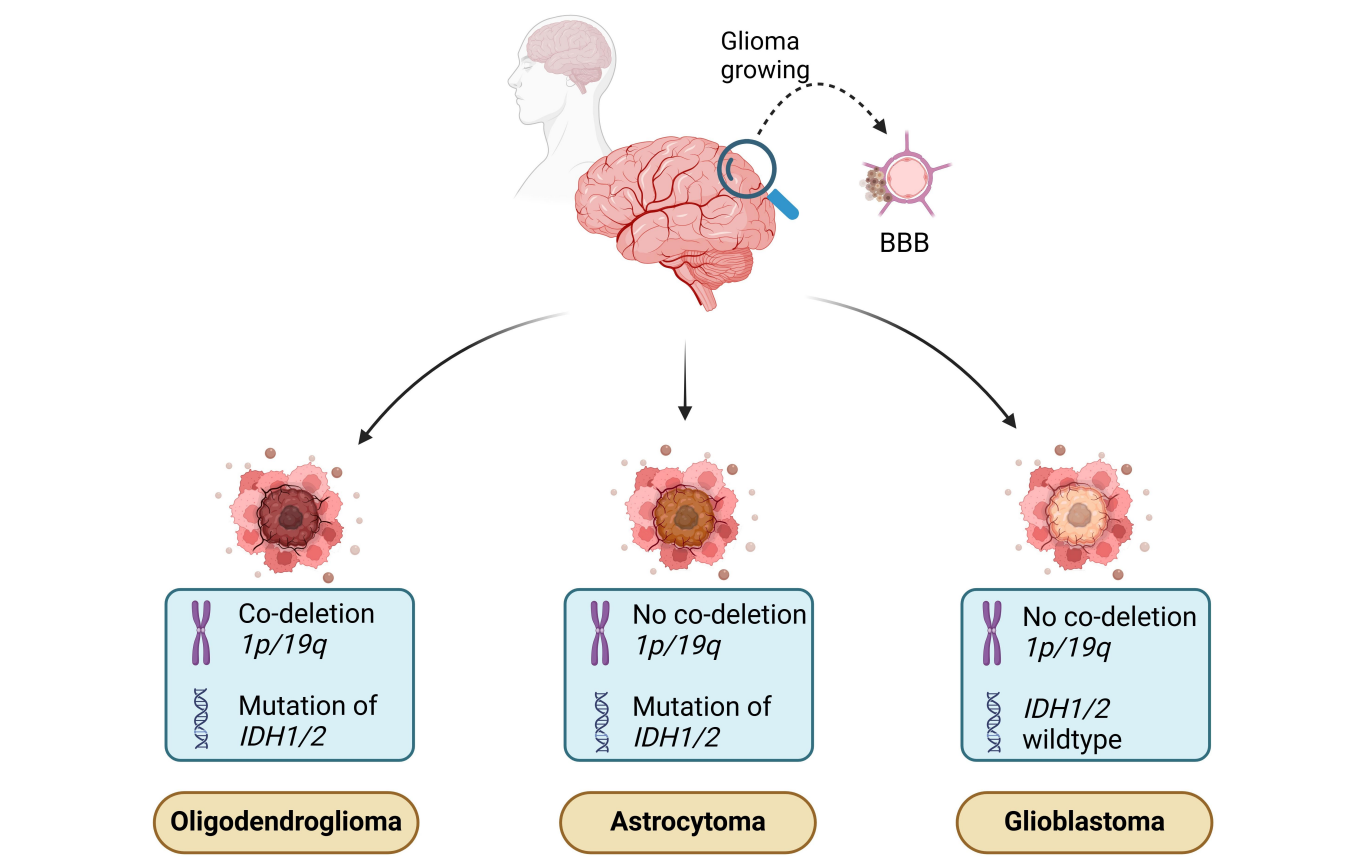
**Molecular and genetic features of malignant gliomas.** The illustration shows three major types of gliomas: oligodendroglioma, astrocytoma, and GBM—based on specific molecular and genetic markers. Oligodendrogliomas are characterized by co-deletion of chromosomal regions *1p/19q* and mutations in the *IDH1/2* genes. Astrocytomas exhibit *IDH1/2* mutations but lack the *1p/19q* co-deletion. GBMs, on the other hand, are *IDH* wildtype and do not have *1p/19q* co-deletion, making them the most aggressive type. These molecular signatures play a key role in the diagnosis and prognosis of glioma subtypes. BBB: blood-brain barrier; GBM: glioblastoma; *IDH1/2*: isocitrate dehydrogenase 1 and 2 genes. Created in BioRender. Ibarra, L. (2025) https://BioRender.com/i19u738

Since 2021, the classification of gliomas has undergone significant changes, with the WHO proposing a new system based on molecular alterations, including *IDH1/2* mutation status and *1p/19q* co-deletion status. This updated classification system categorizes diffuse gliomas into three distinct types: oligodendroglioma with *IDH1/2* mutation and *1p/19q* co-deletion, astrocytomas with *IDH1/2* mutations but without co-deletion, along with *TP53* mutations and *ATRX* loss; and GBM with wild-type *IDH1/2*. These new categories replace the previous distinction between primary and secondary GBM and also exclude gliomas with *IDH* mutations from the GBM group (Figure 1) [26].

The standard clinical treatment for patients with gliomas, regardless of their classification, typically involves an initial surgical resection followed by ionizing radiation therapy, with doses of approximately 60 Gy. However, this treatment often yields suboptimal results, as tumors frequently recur, and RT commonly causes toxic effects on neural tissue. Consequently, adjuvant CTX is employed, utilizing alkylating agents to enhance patient survival [23, 25].

Advancements in molecular technologies have also facilitated the identification of key molecular markers, such as the methylation of the *MGMT* gene promoter, which predicts the response to alkylating CTX agents like temozolomide (TMZ). These markers enable the personalization of treatments and the selection of more effective therapies for patients, thereby improving clinical outcomes. MGMT is a DNA repair enzyme that can restore O-6-methylguanine during genomic damage caused by alkylating agents used in CTX [25]. Consequently, patients with methylated *MGMT* gene promoters are less likely to effectively repair the damage caused by TMZ, making them more responsive to the treatment [17]. In addition, *IDH*-mutant gliomas may respond differently to conventional therapies. For instance, they may exhibit increased sensitivity to certain chemotherapeutic agents, and the mutation status can guide the selection of more effective treatment regimens. For this reason, the unique metabolic and molecular characteristics of *IDH1/2*-mutant gliomas present opportunities for targeted therapies [27]. Ongoing research is being conducted on drugs that are particularly engineered to block the *IDH1/2* enzymes or to offset the effects of 2-HG [3]. These medications possess the capacity to offer novel therapeutic options for individuals impacted by these illnesses. The *IDH* mutation status is commonly used as a criterion for classifying patients in clinical trials, which affects the development and evaluation of new therapies. Evaluating the *IDH* mutation status in gliomas is crucial for determining its suitability for experimental therapies and for reviewing clinical trial outcomes.

In this regard, Tumor-treating fields (TTFields) therapy is recognized as an effective adjunctive treatment for newly diagnosed patients with GBM [28]. In these malignancies, TTFields are low-intensity, intermediate-frequency alternating electric fields with a frequency of around 200 kHz, and the electric field strength typically remains below 5 V/cm in most brain areas [29]. Recent long-term survival results from a prospective study on TTFields have shown significant enhancements in overall survival (OS) and progression-free survival (PFS) when combined with adjuvant TMZ, compared to TMZ alone, in newly diagnosed GBM, integrated into standard therapy, without negatively impacting quality of life [30]. Some retrospective investigations have begun the assessment of the correlation between TTFields and the molecular characteristics of glioma malignancies, including *IDH* mutational status. Nonetheless, the majority of these investigations remain in initial stages, with limited outcomes. A study conducted by Zhang et al. [31] revealed that OS exhibited no significant relation with age, gender, Karnofsky performance status (KPS) score, *MGMT* status, *IDH* status, *EGFR* status, *1p/19q* deletion status, or *TERT* promoter status.

In contrast to conventional and adjuvant treatments, PDT is a non-invasive treatment that employs a PS, light, and endogenous molecular O_2_. Although these components are individually non-toxic, when the PS is exposed to light, ROS are generated, including superoxide anion and/or singlet O_2_ (^1^O_2_), which induce cytotoxicity and cell death. PDT was explored in the context of GBM management with some encouraging results. However, many of these clinical studies have not gathered sufficient information to provide reports on the success or failure of PDT with different PSs such as Photofrin and 5-ALA (NCT01966809, NCT00118222, NCT00002647). Recently, the PS 3-(1-Butyloxy)ethyl-3-deacetyl-bacteriopurpurin-18-n-butylimide methyl ester, commercially known as Photobac, has entered phase 1 clinical trials in the recruitment stage (NCT05363826) as an adjuvant to resection of GBM. Similarly, 5-ALA HCl is in phase 1 clinical trials and the recruitment stage (NCT05736406) for GBM treatment is followed by standard therapy. A completed clinical study (NCT03048240) yielded promising results using 5-ALA HCl in combination with standard treatment, demonstrating increased survival without adverse effects, toxicity, or mortality. The study reported a median OS of 23.4 months.

In the following sections, we will review and discuss the mechanistic aspects, main advantages, and disadvantages of this therapeutic approach applied to malignant gliomas, as well as potential combinations that have been explored with this photo-assisted therapy.

## PDT: basics and main features for GBM treatment

PDT is a medical technique that uses light sources to trigger the activation of PSs for the treatment of cancer and other disorders. This approach is non-surgical in most of the cases and minimally invasive. PDT has received regulatory approval for the treatment of various cancers in several countries, though its specific use for gliomas is still under clinical investigation. In the United States, the FDA has approved PDT for cancers such as non-melanoma skin cancer, esophageal cancer, and non-small cell lung cancer, using PSs like Photofrin. While PDT for gliomas has not yet received formal FDA approval, ongoing clinical trials are evaluating its potential for treating GBM (NCT05736406, NCT05363826). Similarly, the European Medicines Agency (EMA) has approved PDT for certain cancers with glioma-specific applications currently in research. Japan, a leader in PDT innovation, has approved its use for cancers such as lung and gastric cancers, and like the U.S. and Europe, is investigating PDT’s efficacy for malignant gliomas. With the advancement of clinical research worldwide, it is expected that PDT would soon be established as a crucial therapy choice for aggressive brain cancers such as GBM as an adjuvant therapy.

PDT offers a promising approach for treating malignant gliomas by using light-sensitive compounds to generate massive ROS that selectively target and destroy tumor cells [32]. This minimally invasive technique allows for precise control of treatment, reducing damage to surrounding healthy brain tissue, and shows potential in overcoming the challenges of conventional therapies, such as drug resistance and limited efficacy in highly aggressive tumors like gliomas.

A PDT protocol for glioma treatment involves selecting a PS, such as porphyrin-based compounds or prodrugs, that preferentially accumulates in glioma cells. After oral, systemic, or local administration, a waiting period allows the PS to clear from healthy brain tissue. The tumor o remaining tumor cells after surgery are thereafter illuminated with light of a specified wavelength, carefully selected light intensity, usually employing red or near-infrared radiation (NIR), to trigger the activation of the PS [33]. Real-time monitoring and imaging guide the treatment, and post-treatment care includes monitoring for side effects and assessing tumor response [34]. One of the benefits of PDT is its adaptability, as it can be administered in either a single or multiple sessions, dependent on the tumor’s characteristics.

An ideal PS must possess several essential attributes. It should be readily accessible from widely available precursors and demonstrate high quantum efficiency for ^1^O_2_ production (ΦΔ). The absorption range for ideal PS must be between 680 and 800 nm, accompanied by a high molar extinction coefficient (ε max). Another characteristic is that PS must efficiently accumulate in cancer cells while exhibiting little dark toxicity in other organs and tissues. Additionally, it should be easy to administer, soluble in body fluids, and readily eliminated from it.

**Figure 2:**
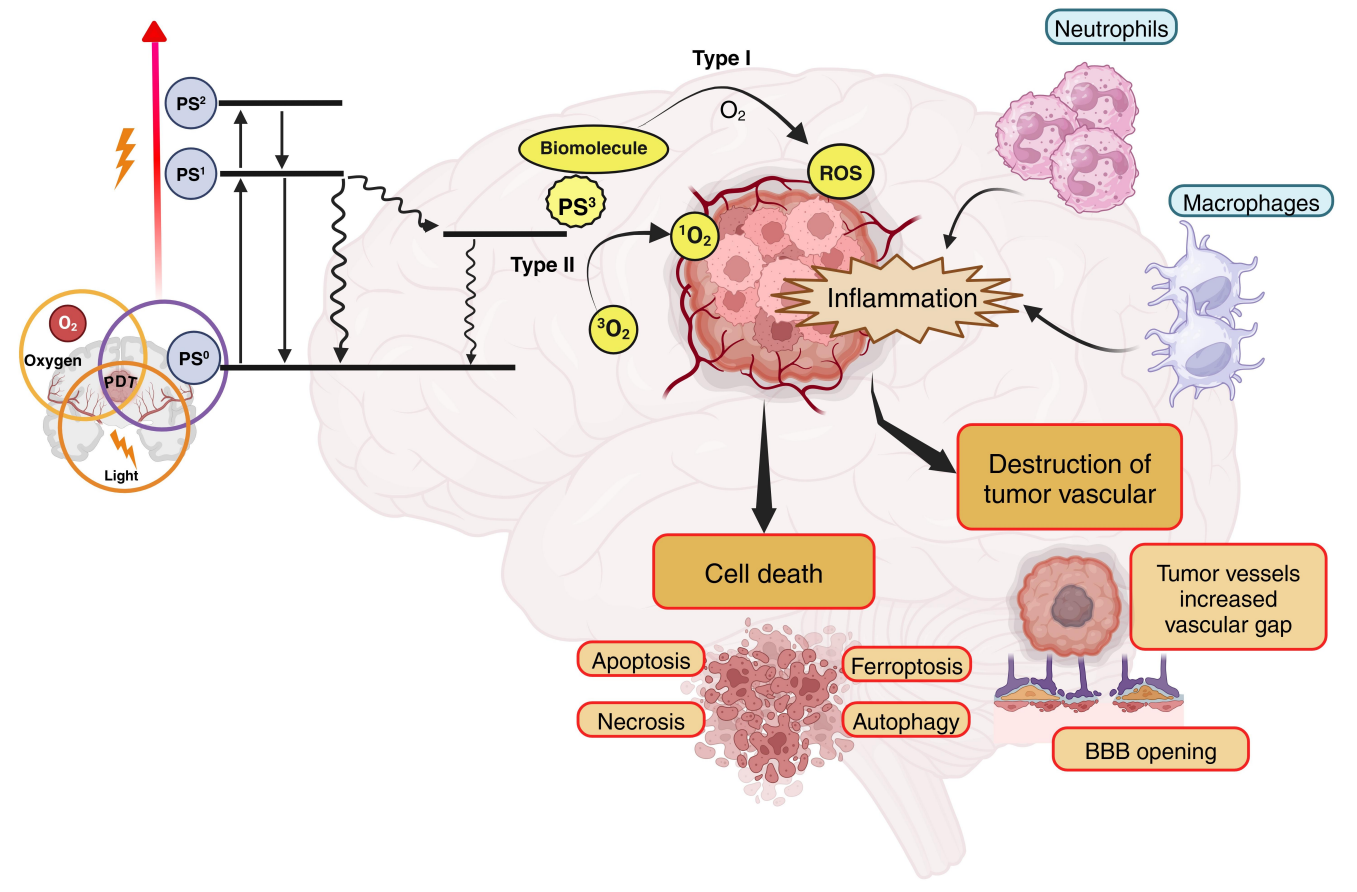
**Mechanism of photodynamic therapy (PDT) in glioblastoma (GBM).** Upon light activation, the photosensitizer (PS) transitions from its ground state (PS^0^) to a short-lived first excited singlet state (PS^1^). The PS^1^ can return to PS^0^ by emitting a photon (fluorescence) or through internal conversion. Alternatively, the PS^1^ can transition to an excited triplet state (T_1_ or PS^3^) via a process known as intersystem crossing. The PS^3^ state has a sufficiently long half-life to participate in subsequent chemical reactions, meaning that photodynamic action is mostly mediated by the PS in this energetic state. The PS^3^ can react with a substrate (water or biomolecule) by donating or receiving electrons and/or abstracting a hydrogen atom, thereby generating radical ions or neutral radicals. These radicals, in turn, can react with oxygen (O_2_) to produce reactive O_2_ species (ROS) either directly or indirectly, such as superoxide anion radical (O_2_•−), hydrogen peroxide (H_2_O_2_), hydroxyl radical (HO•), peroxyl radical (ROO•), alkoxyl radical (RO•), and others. In the presence of O_2_, type II reactions lead to the generation of singlet O_2_ (^1^O_2_) by energy transfer. These processes trigger inflammation, destruction of tumor vasculature, and various cell death mechanisms, including apoptosis, necrosis, ferroptosis, and autophagy. The breakdown of tumor vessels increases vascular gaps and facilitates the opening of the BBB, further enhancing treatment efficacy. Inflammatory responses also recruit immune cells such as neutrophils and macrophages, contributing to tumor destruction. Created in BioRender. Ibarra, L. (2025) https://BioRender.com/d72i758

PDT can eradicate tumor cells via three established mechanisms: (a) direct cellular destruction of tumor cells through different types of cell death, (b) vascular impairment involving blood vessel destruction, alterations in barrier function, constriction of arteriolar vessels, thrombus formation, and stasis of blood flow, and (c) inflammation coupled with the activation of the immune response (Figure 2). The anti-vascular function is interesting to discuss if it is truly a desired mechanism of PDT in this type of tumor. It will be discussed later in the review.

ROS and ^1^O_2_ generated in the final stages of PDT process are highly cytotoxic, triggering mainly apoptotic, necrotic, or autophagy-related cell death mechanisms in tumor cells through oxidative processes. The extent and type of cell death depend on factors such as the subcellular localization of the PS, the oxidative stress intensity, and the tumor microenvironment (TME). Protein and membrane damage play a key role in optimizing the cytotoxic efficiency of PDT. When PDT-induced oxidative stress is moderate, mitochondria-mediated apoptotic pathways are activated. ^1^O_2_ and ROS damage mitochondrial membranes, leading to cytochrome c release and activation of caspases, such as caspase-9 and caspase-3. This process is observed in GBM cells treated with PS that preferentially localize in mitochondria, such as Photofrin or porphyrin-based compounds [35]. At higher oxidative stress levels, excessive ROS production disrupts cellular homeostasis, leading to rapid ATP depletion and plasma membrane rupture. This form of cell death is common in PDT regimens with high fluence rates or in cells with defective apoptotic pathways [36, 37]. Studies in GBM models show that necrotic death is often associated with oxidative damage to proteins [38].

PDT can activate autophagy as either a pro-survival or pro-death pathway, contingent upon ROS levels. In GBM, sublethal oxidative stress stimulates autophagic flux, resulting in the destruction of impaired organelles and proteins. Excessive autophagy can lead to autophagic cell death, marked by the buildup of autophagosomes and the lysosomal destruction of vital cellular components. This has been shown in GBM cells subjected to Hypericin (HYP) PS [39].

Currently, studies on new mechanisms of cellular damage with different PS that had already reported inducing a type of cell death continue to be conducted, demonstrating the triggering of new mechanisms of cellular damage and new pathways of cell death [40–42]. In the case of ferroptosis, phospholipid alterations result from lipid peroxidation, a process initiated by the generation of free radicals and ^1^O_2_. Once initiated, this mechanism becomes autocatalytic, leading to the formation of hydroperoxides and other byproducts [43, 44]. This oxidative lipid damage is particularly relevant, as it leads to the accumulation of lipid peroxides beyond the capacity of cellular antioxidant systems, such as glutathione (GSH) peroxidase 4 (GPX4), to neutralize them. In ferroptosis, these oxidative processes drive the peroxidation of polyunsaturated fatty acids (PUFAs), a key step in the loss of membrane integrity. Irreversible damage occurs when a hydrogen atom is abstracted from an unsaturated fatty acid or lipid with hydrogen (LH), generating a lipid radical (L•), which subsequently combines with an O_2_ molecule to form a peroxyl radical (LOO•). This radical can further react with another LH fatty acid, initiating a new oxidation cycle that generates additional lipid hydroperoxide (LOOH) and L• [45]. During the propagation phase, the LOO• initiates new oxidation chains, while lipid hydroperoxides decompose into other intermediate radicals. In light-induced reactions, alkoxides are generated through direct contact between the triplet-state PS, lipid double bonds, and lipid hydroperoxides, leading to chain fragmentation by cleavage. The accumulation of truncated lipid aldehydes and other oxidation products exacerbates lipid membrane destabilization, triggering membrane rupture and subsequent cell leakage, hallmarks of ferroptosis. Ultimately, this uncontrolled lipid peroxidation overwhelms cellular repair mechanisms, culminating in iron-dependent oxidative cell death, which is a defining feature of ferroptosis [2, 46]. This offers opportunities based on the research conducted by Xu et al. [47], who developed nanocomposites (NC) that can traverse the BBB and specifically target GBM by adhering to an albumin-binding receptor that is highly expressed in both the BBB and GBM. Upon laser irradiation, the ROS-generating PS HYP, encapsulated within the NC, interacts with the ferroptosis inducer erastin to elicit a synergistic anti-GBM effect through PDT and ferroptosis, therefore suppressing GBM proliferation via excessive ROS generation.

**Figure 3:**
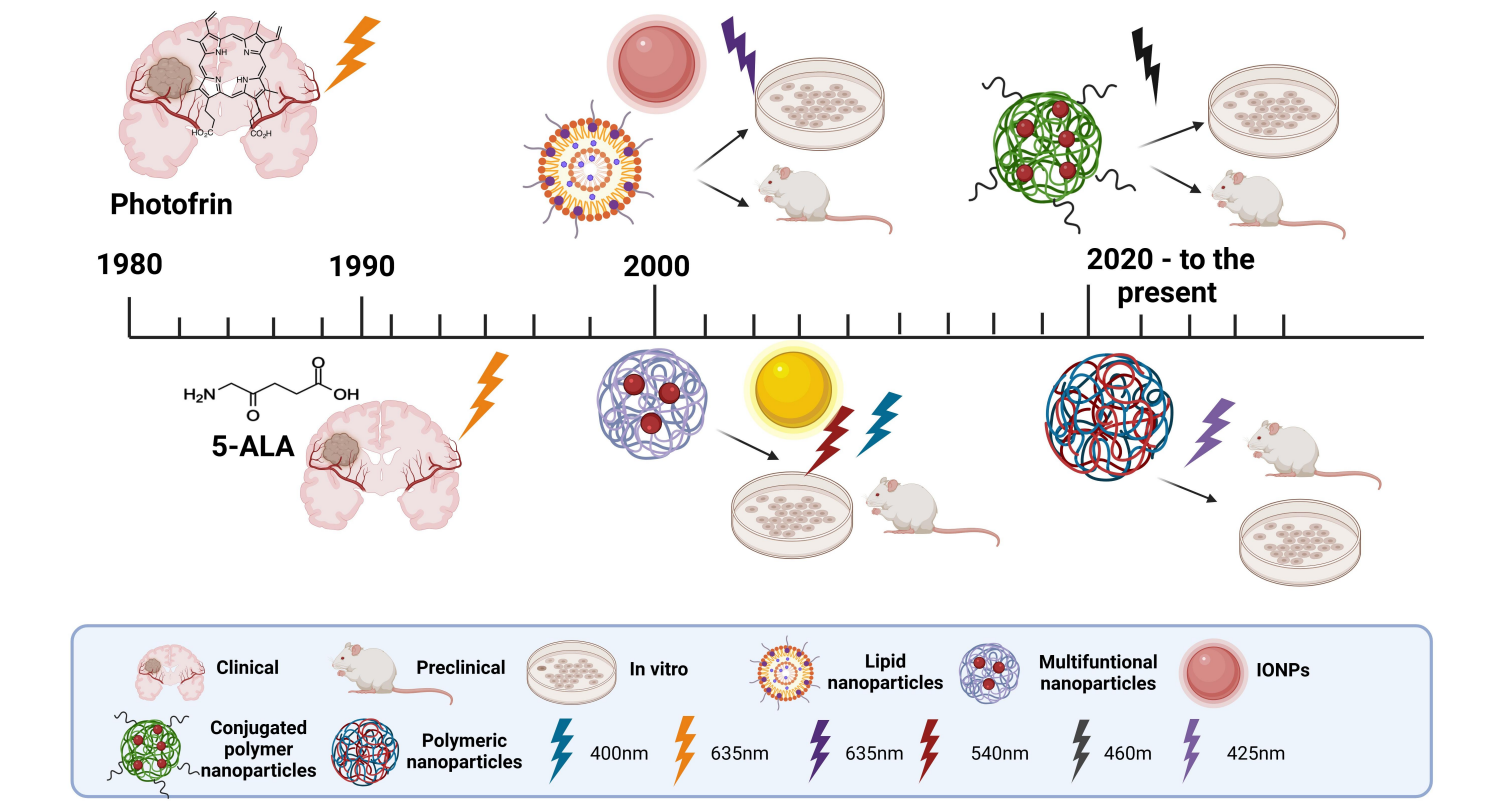
**Overview of photosensitizer (PS) in photo-assisted therapies for gliomas in various stages of research and development.** The image illustrates different types of PS from the first, second, and third generation used in clinical, preclinical, and in vitro studies, highlighting their applications in glioma treatment. Nanoparticle types include conjugated polymer nanoparticles, polymeric nanoparticles, lipid nanoparticles, multifunctional nanoparticles, and iron oxide nanoparticles (IONPs). Light activation wavelengths are shown for different PSs, including 400 nm, 425 nm, 460 nm, 540 nm, and 635 nm, representing the excitation required for PDT in these systems. 5-ALA: 5-aminolevulinic acid. Created in BioRender. Ibarra, L. (2025) https://BioRender.com/g70f990

Over the years, different generations of PSs have been developed (Figure 3), each addressing the limitations of their predecessors. First-generation PSs were the pioneers in clinical PDT applications but were hindered by suboptimal pharmacokinetics and low ΦΔ. In response, second-generation PS was introduced, designed with improved chemical properties, such as higher selectivity and better tissue penetration. More recently, third-generation PSs have been developed, integrating features like targeted delivery and multifunctional capabilities to enhance both therapeutic efficacy and safety. In the following section, the different PSs explored in the context of malignant gliomas will be described.

While both normal and malignant cells can produce ROS, the concentration of some PS, particularly third-generation nano-based PS, is generally much higher in tumors due to the EPR effect. This process enables PS to persist in the afflicted tissue for an extended period, hence decreasing their presence in healthy cells and mitigating adverse effects [48–50]. Moreover, the coupling of PS with NPs and other ligands, including monoclonal antibodies (mAbs), enhances its distribution and precise targeting to malignant cells [12]. Due to the elevated or differential expression of antigens and receptors in GBM cells, PS engineered to identify these biomarkers can be selectively internalized through endocytosis. This facilitates the formation of ROS primarily in tumor cells upon light activation, leading to their demise while preserving adjacent healthy tissue. Additionally, the use of nanotechnology in the delivery of photodynamic drugs enhances the pharmacokinetics and biodistribution of PS, thereby increasing their therapeutic efficacy. Advanced strategies, such as the conjugation of PS with NP targeting specific tumor receptors, further improve selective accumulation in GBM cells, ensuring a more precise and effective treatment.

### First-generation PSs in GBM

First-generation PSs include natural porphyrins such as HP and its purified derivatives, such as sodium porfimer (Photofrin) [51]. Porphyrins are aromatic compounds consisting of four pyrrole rings joined by methine bridges. They have unique optical properties, such as a complex absorption spectrum due to their electronic structure and a fixed absorption wavelength in the visible region. Their absorption spectrum lies in the 380–500 nm region with high intensity, and the Q-bands, located between 500 and 750 nm, have lower intensity. Most of these PSs, due to their lipophilic nature, need to be encapsulated in nanocarriers [52]. The use of HpDs in malignant neoplasms was first reported in the early 1960s, and their application as PSs in gliomas began about a decade later. By the 1980s, first-generation PS had been clinically tested for the treatment of malignant gliomas in several countries, including Italy, Australia, and the United States, with reports of successful therapy. Despite these promising outcomes, the chemical properties of these compounds limit their efficacy as ideal candidates for PDT. Specifically, they exhibit low therapeutic efficiency and a low ΦΔ [53]. While these compounds demonstrate strong absorption around 400 nm, their absorption at longer wavelengths is less strong and even limited [54].

### Second-generation PSs in GBM

Second-generation PSs exhibit greater purity, enhanced efficiency in ROS production, and improved tumor selectivity, while reducing adverse effects. These PS include porphyrin-based structures and precursors, such as 5-ALA, temoporfin, boronated porphyrins, benzoporphyrin derivatives, and chlorins like sodium talaporfin. Second-generation PS possesses phototoxic properties at longer wavelengths (600–800 nm) and can be excited with lower energies (up to 20 J/cm^2^), allowing for deeper penetration into tumor tissues. Over the past three decades, these PS have been clinically evaluated for the treatment of gliomas [54–57]. Additionally, other second-generation PS, such as metallophthalocyanines like chloro-indium-phthalocyanine [58], have been extensively studied for brain tumors in both pediatric and adult patients [53].

5-ALA is one of the most commonly employed PS for the treatment of malignant gliomas. As a non-photoactivatable prodrug in PDT, 5-ALA is metabolized into protoporphyrin IX (PpIX) through the heme biosynthesis pathway, which naturally occurs in the mitochondria from glycine and succinyl CoA. When exogenous 5-ALA is administered in excess, PpIX accumulates within the mitochondria because the biosynthetic conversion to heme, necessary for its removal, is limited [59]. Neoplastic tissues, in particular, produce an excess of PpIX compared to surrounding normal tissues. This selective accumulation is due to the fact that malignant tissues exhibit reduced ferrochelatase activity (the enzyme responsible for converting PpIX to heme), while the activity of porphobilinogen deaminase (PBG-ase), an enzyme involved in heme synthesis, is increased [32]. This imbalance favors the selective accumulation of PpIX in tumor cells, creating a differential gradient of protoporphyrin concentration between normal and malignant tissues. Consequently, the active PS accumulates predominantly within tumor cells, enabling PDT to selectively target and destroy these cells [60]. Nonetheless, the ability of 5-ALA to produce elevated levels of ROS is constrained, presenting a drawback to its therapeutic effectiveness. The photoactivation of 5-ALA occurs primarily at a wavelength of 635 nm [61]. However, some studies have also demonstrated that the PS can achieve excitation through two-photon excitation (TPE). TPE involves the simultaneous absorption of two photons, with each photon contributing half of the energy required to excite the PS. This method offers advantages such as enhanced light penetration in tissues, which is beneficial for deeper therapeutic effects [62].

One limitation of 5-ALA is its zwitterionic and hydrophilic nature at physiological pH, which restricts its ability to cross cellular biological barriers [63]. Based on this knowledge, attempts have been made to demonstrate the ability of ALA to cross the BBB and its selectivity towards tumor cells in the brain parenchyma, as it is a prodrug used clinically for photodiagnosis [64]. Studies have shown that it crosses the BBB to a limited extent via passive diffusion, although with a slow influx rate constant [65]. A recent immunofluorescence examination in patients using antibodies that target various components of the BBB indicated that 5-ALA does not penetrate the intact BBB, notwithstanding its diminutive size. The fluorescence of PpIX caused by 5-ALA is well-established in high-grade glioma (HGG) surgery with a disrupted BBB, but its utility is constrained in low-grade glioma surgery, particularly when the BBB remains intact [66].

Furthermore, tumor hypoxia may exacerbate during PDT, perhaps due to O_2_ consumption for ROS formation or indirectly through impaired blood flow in tumor vasculature resulting from endothelial injury. This reduction in O_2_ supply represents a significant obstacle to the efficacy of 5-ALA-based PDT, limiting the generation of cytotoxic ROS and, consequently, its full therapeutic potential [67].

An intriguing study by Jones et al. [68] demonstrated that glioma cells previously exposed to 5-ALA release extracellular vesicles (EVs) that carry disease fluorescence biomarkers, such as PpIX-incorporated PS. The researchers evaluated both animal and human models to explore whether 5-ALA-treated glioma cells, in vitro and in vivo, release PpIX-positive EVs into the circulation. These EVs were captured and analyzed, highlighting the potential of plasma-derived, PpIX-positive EVs as a diagnostic tool for malignant gliomas. This approach presents a novel liquid biopsy platform for confirming and monitoring tumor status, offering a less invasive method for diagnosis and disease tracking and validating PpIX positive EVs crossing BBB, which were first developed into glioma cells.

### Third-generation PSs in GBM

In the early 2000s, in vitro studies on PDT for GBM focused on third-generation PSs, which offered greater local specificity or selectivity, improved cellular internalization, and more efficient retention of the PS [53]. These third-generation PSs are made from or employ various delivery vehicles, including polymer- or lipid-based NPs, liposomes, organometallic complexes, albumin- or antibody-conjugated nanospheres and nanocapsules, micelles, dendrimers, nanocrystals, and gold NPs. Currently, no third-generation PSs have received approval for clinical application in PDT for humans.

Nanomaterials can be synthesized from a wide variety of both natural and synthetic materials. NPs are relatively easy to fabricate, facilitating the targeted delivery of various agents. NPs can be used as PS due to several factors such as high surface-to-volume ratio. Their large surface area relative to volume allows for significantly enhanced delivery of PS to target cells [69]. In a recent study, chlorophyll α derivative named pyropheophorbide α 17-diethylene glycol ester (XL) was encapsulated in poly(lactic-co-glycolic acid) (PLGA) NPs increasing XL solubility and selective tumor-targeted accumulation [70]. Encapsulation within NPs can protect the PS from premature degradation or inactivation by biological components such as blood plasma proteins. This prolongs the PS’s stability and ensures its accumulation in tumor tissue [71]. Furthermore, the optimal PS has amphiphilic characteristics, indicating solubility in both aqueous and lipid environments, possesses minimal dark toxicity and significant photocytotoxicity, maintains stability during storage, and is economically viable [70]. PS-loaded NPs may be efficiently delivered via the circulatory system to the tumor site, and for malignant gliomas, these PSs facilitate traversal of the BBB to target GBM cells [72, 73]. To optimize biological distribution, pharmacokinetics, cellular uptake, and NP targeting, various functional groups or targeting fragments can be added to the particle surface to increase tumor active targeting [12, 74].

Different second-generation PSs with notable photodynamic capabilities have been encapsulated to enhance specific suboptimal qualities, resulting in the creation of several NP-based delivery systems for these molecular drugs. Conversely, a distinct category of nanoparticulate materials has arisen as third-generation PS, which could not be utilized as conventional molecular PS. We shall delineate several pertinent examples from recent preclinical experiments. The incorporation of nanocarrier systems in PDT aims to improve the selectivity and efficacy of treatment. These systems facilitate targeted delivery of PS to the tumor, enhancing selective accumulation and reducing systemic side effects.

#### Inorganic NPs

Titanium oxide (TiO_2_) NPs serve as PS that, upon ultraviolet (UV) light irradiation, generate ROS, leading to tumor cell death. Studies have demonstrated their efficacy in reducing glioma cell viability in vitro [75, 76]. Gold NPs, known for their unique optical properties, can be functionalized with PS to enhance tumor targeting, generating both heat and ROS upon irradiation to induce apoptosis [77]. Additionally, quantum dots, semiconductor nanocrystals, offer potential for PDT activation and biomedical imaging, further advancing NP-based PDT approaches [78].

#### 5-ALA-loaded NPs

5-ALA is a recognized prodrug utilized in PDT for gliomas due to its capacity to preferentially stimulate the synthesis of PpIX. However, its clinical efficacy is often limited by challenges such as low bioavailability and poor accumulation at the tumor site. To overcome these limitations, recent advancements have focused on incorporating 5-ALA into NP-based delivery systems [79]. One promising approach involves loading 5-ALA into periodic mesoporous organosilica (PMO) NPs, coated with Prussian blue (PB), which enhances cellular uptake, increases PpIX production, and improves oxygenation at the tumor site, thereby boosting the overall efficacy of PDT. These systems not only offer enhanced biocompatibility but also address key challenges such as the decomposition of hydrogen peroxide (H_2_O_2_) into O_2_, further improving treatment outcomes in gliomas [80].

Biodegradable polymeric NPs are attractive materials for encapsulating PSs, as many have already received FDA approval. For example, anti-PD-L1 antibody, 5-ALA, and magnetic NPs (MNPs) were self-assembled in the presence of biodegradable poly(γ-glutamic acid) (γ-PGA). Subsequently, the bradykinin B1R ligand des-Arg9-kallidin (d-K) was conjugated to the surface to create the final NPs capable of traversing the BBB. Upon excitation with a 980 nm laser, GBM cells underwent apoptosis [81]. The immunogenic cell death (ICD) triggered an inflammatory response that activated the TME, thereby promoting increased infiltration of cytotoxic T lymphocytes (CTLs) into the GBM. These NPs could serve as an effective platform for overcoming barriers in GBM immunotherapy.

#### Berberine-loaded NPs

Berberine (BBR) is an isoquinoline alkaloid found in the Annonaceae and Ranunculaceae families [82]. This natural chemical is traditionally utilized in Chinese medicine and has been shown to cross the BBB, demonstrating advantageous effects in the CNS [83]. Its use in PDT is more recent and experimental [84]. Due to the limited solubility of BBR, its reduced oral bioavailability, and the exocytic activity of P-glycoproteins in cancer cell membranes, the overall therapeutic efficacy of BBR is relatively low. To address these limitations, BBR has been encapsulated into NPs. Hydrophobic salts of BBR, such as dodecyl sulfate (S) and laurate (L), were encapsulated in PLGA-based NPs and coated with chitosan by adding chitosan oleate during preparation. These BBR-loaded NPs efficiently internalize in T98G GBM cells. Among the BBR NPs, BBR-S exhibited the highest efficiency in inducing cytotoxic events, leading to its selection for evaluating the effects of PDT. PDT significantly enhanced the reduction in cell viability of BBR-S NPs at all studied concentrations, achieving an approximate 50% reduction in viability. Importantly, no significant cytotoxic effects were observed in normal primary astrocytes. In GBM cells, a notable increase in both early and late apoptotic events was recorded with the use of BBR NPs, further amplified by PDT application.

#### Conjugated polymer NPs

Conjugated polymers (CPs) and the NPs derived from them (CPNs) represent a promising frontier in PDT due to their unique optical properties and biocompatibility [85, 86]. These polymers exhibit strong light absorption and efficient energy transfer capabilities, which are critical for generating ROS upon light activation [87]. When formulated into NPs, these CPs can enhance the delivery of PSs directly to tumor sites, increasing therapeutic efficacy while minimizing damage to surrounding healthy tissues [88]. Additionally, the ability to functionalize these NPs with targeting ligands allows for selective accumulation in cancer cells, making them a powerful tool for localized treatment in various malignancies, including GBM [12]. As research advances, the potential of CP NPs in PDT continues to expand, paving the way for innovative approaches in cancer therapy. Among different CPs, poly(9,9-dioctylfluorene-alt-benzothiadiazole) (F8BT) was extensively used to synthesize CPNs for bioimaging and PDT applications [89–91]. In order to improve ΦΔ, volumetric doping with platinum octaethylporphyrin (PtOEP) was performed. These systems were developed with the aim of generating ^1^O_2_ in an amplified manner compared to the CP alone and the porphyrin alone [92]. By integrating CPs with traditional PSs, these novel formulations enhance the production of ROS upon light activation, thereby increasing the efficacy of PDT [87]. This synergistic effect not only improves the therapeutic potential against cancer cells but also facilitates more efficient energy transfer and greater light absorption, making these advanced systems a key innovation in the field of PDT [93]. Additionally, poly(styrene-co-maleic anhydride) (PSMA) was incorporated to achieve colloidal stability in high-strength aqueous media of CPNs [12, 36]. CPNs were evaluated at different concentrations in GBM cell lines (U87MG, T98G, and MO59K) using both low-intensity and conventional irradiance light energy. For these types of NPs, the low-intensity irradiance modality was the most efficient to activate it and eradicate GBM cells in vitro and in vivo [36]. A limitation of the application of this type of NPs in the clinic is the lack of rigorous studies regarding their elimination from the body, adverse effects in vivo experiments, and preclinical toxicological studies that validate their therapeutic potential and safety.

#### Graphene-based NP PS

Graphene is a novel nanomaterial that has garnered significant interest in the scientific community owing to its remarkable physical and chemical properties. Graphene-based materials are often developed as smart platforms for nanocarriers and targeted drug delivery. This material possesses delocalized π bonds that account for its distinctive electrical characteristics, enabling graphene to heat under near-infrared irradiation. A significant production of ROS products is also common [94]. Akhavan et al. [95] reported the application of reduced graphene oxide nanoribbons functionalized with amphiphilic polyethylene glycol (rGONR-PEG) and an arginine-glycine-aspartic acid (RGD)-based peptide to target αvβ3 integrin receptors on the human GBM cell line U87MG, exhibiting cytotoxic effects while maintaining low cyto- and particularly geno-toxic effects. Graphene-based NPs enhance electron transfer processes, increasing ROS generation during PDT following covalent and doping interactions with other molecular components [96–99].

Graphene-based nanomaterials offer significant advantages for GBM treatment by integrating PDT and photothermal therapy (PTT) into a single platform. Their exceptional optical properties enable efficient absorption of NIR light, facilitating both the generation of ROS for PDT-induced oxidative stress and localized hyperthermia (HPT) for PTT-mediated tumor destruction. The synergistic combination of PDT and PTT enhances therapeutic efficacy by inducing multiple cell death pathways, overcoming glioma resistance mechanisms such as hypoxia, and minimizing damage to surrounding healthy tissues [97, 100]. This multifunctional approach positions graphene nanomaterials as promising candidates for advanced GBM treatment.

Graphene-based materials have emerged as highly versatile tools in biomedical applications, demonstrating unique capabilities in two distinct domains such as PDT for cancer treatment and electrical stimulation for neuronal differentiation and regeneration [101]. Their multifunctionality arises from their exceptional physicochemical properties, including high electrical conductivity, mechanical strength, biocompatibility, and tunable surface characteristics [102]. These properties may enable graphene-based materials to serve as both PS in PDT and as active components in neural tissue engineering. Different scaffolds incorporating graphene-based nanomaterials were able to induce neuronal differentiation through their unique physical features, such as surface porosity and nanoscale wrinkles, which mimic the extracellular matrix and provide mechanical cues for cell adhesion and proliferation [103–105]. Additionally, these materials can be used as electrodes to deliver electrical stimulation, which has been shown to enhance neuronal differentiation and axon outgrowth [106]. This application does not require light activation and instead focuses on creating a conducive microenvironment for neural tissue repair. However, in the future, a scaffold could be envisioned that allows for the elimination of residual tumor cells after surgical resection and subsequently continues to be used to stimulate neuron proliferation at the affected site. This requires further research.

#### Multifunctional NP-based PS for enhanced therapeutic efficacy

Most recent findings on enhancements in PS design and development for glioma treatment are predicated on multiple attack NPs activated by light. By integrating photosensitizing agents into multifunctional nanocarriers, these systems improve PS solubility, stability, and tumor-targeting capabilities, overcoming limitations such as poor bioavailability and off-target effects. Additionally, NP-based PS can be engineered to facilitate controlled drug release, optimize light absorption, and enhance ROS generation, leading to more effective tumor eradication. Their multifunctionality also allows for the incorporation of imaging agents and therapeutic molecules, enabling real-time tumor monitoring and synergistic treatment approaches [107]. This innovative platform holds significant potential for improving PDT outcomes in GBM, offering a more precise and effective alternative to conventional therapies. The advancement of accurate intraoperative imaging and postoperative residual extraction methods will enable the complete eradication of GBM. Recently, Chen et al. [108] developed a self-disassembling porphyrin lipoprotein-coated calcium peroxide (CaO_2_) NP (PLCNP) to target GBM, facilitating fluorescence-guided surgery (FGS) and enhancing PDT for residual GBM by mitigating hypoxia. The CaO_2_ cores were able to release H_2_O_2_ in the lysosome, which is then degraded into O_2_ providing an important and lacking substrate in GBM. This innovative method may function as an integrated nanotheranostic platform for facilitating accurate GBM resection and enhancing post-operative PDT.

A significant constraint for PDT is the penetration of light required to activate PS. To address this obstacle, the domain of light-activatable nanotheranostics has arisen as a promising approach, utilizing the distinctive characteristics of light to penetrate deeper into brain tissues and kill GBM at the molecular level noninvasively. This factor is especially vital for overcoming the problems presented by the skull as an obstruction in the treatment of GBM. NIR light has attracted considerable interest due to its capacity to enter biological tissues deeply with minimum absorption, facilitating the non-invasive activation of PS nanomaterials. As the wavelength extends further into the NIR spectrum, encompassing NIR-I (650–950 nm), NIR-II (1,000–1,350 nm), and NIR-III (1,550–1,800 nm) biological windows, the depth of tissue penetration increases [109]. Recent findings revealed that europium hexaboride (EuB_6_) NPs, classified as rare-earth boride-based NPs, can sensitize the production of ^1^O_2_ upon NIR-II 1,064 nm photoexcitation and facilitate the generation of hydroxyl radical (HO•) using NIR-III 1,550 nm light. Furthermore, in vivo experiments demonstrated a twofold increase in the average half-life for treated mice compared to the control group in an irradiation protocol without skull injury [110].

A promising and innovative approach recently explored in GBM treatment involves the enhancement of PS nanomaterials through advanced active targeting strategies. One of the most effective methods to achieve this involves the functionalization of NPs with cell membranes and EVs, improving their selectivity, biocompatibility, and therapeutic efficacy. In one direction, the use of cell membrane-coated NPs allows PS nanomaterials to mimic the biological properties of native cells, enhancing their ability to evade immune detection and prolong circulation time. Using cell membranes instead of whole cells in NP functionalization offers several key advantages such as reduced risk of immune response and long-term storage. Different cell types were utilized as cell membrane donors for GBM management, including macrophages [111], neutrophils [112], and tumor cells [113]. These investigations demonstrated the penetration of the BBB and increased accumulation of NPs in GBM cells.

On the other hand, EVs, including exosomes, serve as natural carriers for PS delivery due to their high biocompatibility, ability to cross the BBB, and selective uptake by tumor cells. Coating NPs with EVs involves a combination of biological isolation, chemical modification, and bioengineering techniques to ensure efficient attachment and functional integration [114]. Multiple approaches have been reported for loading therapeutic cargo such as small molecule drugs, including PS into EVs. These approaches can be categorized as pre-loading and post-loading methods. Pre-loading is based on co-incubation or transfection of donor cells with the PS cargo to generate EV-decorated NPs into cells [115]. However, a more uniform and controllable method with greater possibilities for subsequent modifications requires first obtaining the EVs and then fusing or loading them with the desired PS [116, 117].

### Dyes as non-conventional PSs for GBM

Recent research has begun to explore the potential of non-traditional dyes as PSs in PDT, broadening the scope of available agents beyond the commonly used porphyrins and phthalocyanines (Pcs). These alternative dyes, which may include natural pigments, organic dyes, and synthetic compounds, offer unique optical properties and mechanisms of action that can enhance the efficacy of PDT. Their diverse absorption spectra and improved solubility characteristics may facilitate better tissue penetration and targeted delivery, potentially leading to more effective treatment outcomes. As studies progress, these innovative PSs hold promise for overcoming the limitations of conventional agents and expanding the therapeutic options for various malignancies. For example, in order to illustrate the combined impact of acridine orange (AO) and light on 373 MG GBM cell lines, AO was employed as a PS. AO is a non-porphyrinic aniline dye that possesses unique properties in comparison to other PSs. AO is a hydrophobic base with a low molecular weight (265 g/mol), which enables it to swiftly accumulate in lysosomes, enter the cytoplasm, and cross the plasma membrane. This capacity to accumulate in lysosomes, which are characterized by a highly acidic environment, is crucial for the successful process of photosensitization [118]. In this study, AO was able to dramatically induce cytotoxic effects after exposure to 10 minutes in GBM cells in vitro.

Curcumin (Cur), a bioactive polyphenol extracted from the rhizome of turmeric (*Curcuma longa* L.), possesses the capability to successfully penetrate the BBB. The therapeutic efficacy of Cur, both in the absence and presence of blue light, was assessed in T98G GBM cells [119]. Cur demonstrated a photodynamic impact by inducing ROS-mediated apoptosis through the downregulation of the MMP2 and MMP9 pathways. Cur is typically photoactivated at a wavelength of 420 nm, corresponding to the blue region of the visible light spectrum; however, photoactivation using NIR has been shown to enhance cytotoxic and cytostatic effects in GBM cells [38]. NIR irradiation will be more suitable for GBM treatment due to its superior tissue penetration capabilities.

Methylene blue (MB), an FDA-approved drug used to treat conditions such as cyanide poisoning, carbon monoxide poisoning, and methemoglobinemia, has shown promise as a PS for PDT in the treatment of gliomas. In the context of brain diseases, controlling angiogenesis and maintaining BBB permeability presents a significant challenge, which may be addressed by regulating the metabolic status of cerebral endothelial cells (CECs). Such regulation directly influences angiogenic mechanisms and BBB permeability within the glioma microenvironment. In vitro studies have shown that the application of MB or laser irradiation by itself induces a shift in the redox status of CECs towards increased reducing activity, without causing cell damage. However, the combined application of MB and laser radiation exerts the opposite effect, leading to an increase in the oxidized form of the FAD coenzyme and ultimately resulting in cell death [120].

## Clinical investigations concerning PDT treatments for malignant gliomas

PDT has emerged as a promising adjunctive treatment for malignant gliomas, with several clinical trials conducted worldwide. The contributions of research groups from Japan, Germany, Scotland, Canada, and Austria stand out. The contributions of Stummer’s German group to PDT in malignant gliomas are significant, particularly through their pioneering work on the use of 5-ALA for FGS. Their landmark study demonstrated that intraoperative fluorescence could enhance the resection of HGGs by clearly distinguishing tumor tissue from healthy brain tissue, which is crucial for improving surgical outcomes [121]. Stummer and his team conducted a phase III clinical trial that highlighted the benefits of 5-ALA-induced fluorescence, showing that it significantly increased the extent of tumor removal compared to conventional techniques [122]. The study reported improved PFS rates among patients treated with fluorescence-guided resection (FGR), establishing a new standard of care for glioma surgeries. Additionally, their research has explored the combination of PDT with CTX, further enhancing the efficacy of treatment regimens for malignant gliomas [122, 123]. This group examined the potential of PpIX as a serum marker for HGGs to monitor recurrence [124]. Patients [HGG: *n* = 23 pediatric (pHGG); *n* = 5 recurrent (rHGG)] who underwent FGR received 5-ALA following the standard clinical procedure. The control group of eight healthy volunteers (HCTR) also received 5-ALA. Serum was collected before and repeatedly up to 72 h after drug administration. A significant accumulation of PpIX in HGG was observed following 5-ALA administration [analysis of variance (ANOVA): *p* = 0.005, post-hoc: HCTR vs. pHGG *p* = 0.029, HCTR vs. rHGG *p* = 0.006]. Baseline PpIX levels were similar between patient and control groups. Therefore, 5-ALA is required for PpIX induction, which is safe at the standard clinical dose. PpIX may be a novel target for liquid biopsy in gliomas, so larger clinical studies are required to fully characterize its potential.

Another study by Pepper et al. [125], in which this PS was used, was the one in phase I/II conducted at the University Hospital of Münster, which evaluated the combination of oral 5-ALA and RT in patients with recurrent GBM. Thirty patients over 18 years old with histologically confirmed recurrent supratentorial GBM and good functional status (KPS ≥ 60) were recruited. Following a 3 + 3 dose-escalation design, patients who underwent repeat resection received RT at a dose of 36 Gy and fractions of PDT, which included oral 5-ALA administration before irradiation sessions. The study aimed to determine the maximum tolerated dose (MTD) of repeated 5-ALA administration, gradually increasing number of PDT fractions from one to eight, while closely monitoring safety and toxicity. Two cohorts received neoadjuvant treatment fractions before surgery. Follow-ups were conducted two and five months after treatment. The primary endpoints included the MTD, while secondary endpoints were event-free survival, PFS, and OS. Additionally, 5-ALA metabolites and tissue effects in resected samples were analyzed.

Despite the lack of improved outcomes with various other treatments, the application of 5-ALA as a radiosensitizer shows significant promise owing to its preferential absorption by glioma tumor cells, hence preserving normal brain tissue. Furthermore, 5-ALA is simple to administer and is typically well tolerated, as evidenced in the context of surgical procedures and PDT. This experiment was established to address recurrent GBM, due to the significant demand for effective salvage treatment alternatives in this patient population and the absence of a definitive treatment standard. In the event of favorable outcomes, additional assessment may be contemplated as an adjunct to conventional first-line therapy. Nevertheless, data regarding subsequent drug applications are not currently accessible. In the realm of brain tumor surgery, single doses of up to 60 mg/kg have been documented as well tolerated, thereby warranting a dose-escalation experiment with increments of 20 mg/kg. The researchers assert that the results from this inaugural single-center dose-escalation study in humans may provide a crucial instrument for treating GBM patients and herald a new era of enhanced RT efficacy against this highly aggressive tumor type.

The Japanese group of Kaneko et al. [126] conducted clinical trials concentrating on PDT for malignant gliomas. Their strategy comprised giving PSs such as 5-ALA before tumor excision and delivering intraoperative PDT by light diffusers or balloon devices inserted in the resection space. These trials indicated moderate improvements in survival, with PDT suggesting potential for better outcomes when coupled with surgical resection [127]. Another study assessed a different PS, talaporfin sodium, in a multicenter clinical trial investigating the efficacy and safety of intraoperative PDT utilizing talaporfin sodium in patients with primary malignant brain tumors, specifically GBM. The trial had 22 patients, demonstrating a 12-month OS rate of 95.5% and a 6-month PFS rate of 91%. The treatment was well tolerated, with adverse effects similar to standard neurosurgical practices, and no significant complications directly associated with the PDT [128]. However, the studies underlined the need for revised methods and further studies to enhance efficacy and safety of GBM treatment.

The Innsbruck group, under Kostron’s leadership, performed clinical experiments indicating that PDT with HpD can improve survival rates in patients with malignant gliomas [129]. The research demonstrated enhanced PFS and OS relative to conventional therapy. Patients exhibited little occurrence of serious adverse effects, reinforcing the safety and usefulness of PDT as a supplementary treatment to traditional therapies. The findings suggest that PDT may serve as a viable approach for the treatment of malignant gliomas. Nonetheless, the outcomes of cavitary PDT with HpD are promising, yet require a comprehensive phase III investigation. Another study investigated 5,10,15,20-tetrakis(3-hydroxyphenyl)chlorin (mTHPC)-mediated photodynamic diagnosis (PDD) and PDT for malignant brain tumors, notably a phase-II trial including 22 patients, which demonstrated that FGR utilizing mTHPC improves tumor detection and excision [130]. The study indicates a sensitivity of 87.9% and specificity of 95.7% for tumor detection, proposing that the integration of intraoperative visualization with therapy presents a promising approach to enhance surgical outcomes in brain cancer treatment.

Eljamel, a prominent Scottish researcher, has made significant contributions to the field of GBM treatment, particularly in PDT. His work focuses on developing and evaluating PDT as an adjuvant treatment for this challenging disease. Eljamel has conducted multiple clinical studies demonstrating the effectiveness of PDT combined with PSs like Photofrin^®^ in treating GBM. His research has shown that this therapy can extend patient survival, particularly in cases of recurrent GBM. In a prospective study, he evaluated FGR using ALA and Photofrin^®^ alongside repeat PDT sessions in GBM treatment. Twenty-seven patients were recruited (13 in the study group and 14 in the control group), with the study group achieving a median survival of 52.8 weeks compared to 24.6 weeks in the control group. FGR with ALA and Photofrin^®^ and repeat PDT provided a valuable survival benefit without additional risk to patients with GBM [131]. Another study included 73 patients with a median age of 59 years, 30 of whom received PDT and 43 did not. Median survival for patients treated with PDT was significantly higher than for those receiving standard therapy alone (62.9 weeks versus 20.6 weeks). Patients under 65 years of age had longer survival compared to those over 65. Intraoperative RT alone did not yield a significant survival benefit. However, the median survival of patients receiving PDT combined with intraoperative RT was greater than those receiving PDT alone. PDT for GBM was a statistically significant therapeutic modality, and its effects were further enhanced by combining it with intraoperative RT [132].

In another study, twenty patients with malignant brain tumors were administered 20 mg of ALA per kg of body weight orally, three hours before anesthesia. Surgery was conducted using image-guided systems and ALA-induced fluorescence microsurgical techniques. During the procedure, fluorescence intensity was categorized as red, pink, or blue and further measured using a 405 nm pulsed laser and a compact spectrometer with a contact probe placed on the tissue. The extent of tumor invasion was evaluated intraoperatively with standard white light, blue light, and spectroscopic measurements. The study revealed that fluorescence imaging showed GBM to be wider than previously indicated by contrast-enhanced magnetic resonance imaging (MRI), although metastases (MET)-enhanced MRI displayed a similar size to fluorescence imaging. Solid tumors were effectively identified and measured intraoperatively through fluorescence and spectroscopy, allowing for safe resection. Additionally, infiltrative tumor tissue could be identified and removed in non-eloquent brain areas, maximizing surgical outcomes [133].

Dr. Muller and Dr. Wilson’s team [134, 135] at St. Michael’s Hospital in Toronto has investigated PDT for the treatment of malignant brain tumors, particularly GBM and other primary brain tumors such as malignant astrocytoma (MA), malignant mixed glioma, and ependymoma (EP). In their clinical trials, they utilize adjuvant photodynamic treatment following tumor excision, employing a porphyrin-based PS that is injected prior to surgery. During the process, a specific device with an inflating balloon is utilized to administer light, ensuring optimal light distribution within the cavity formed by tumor removal, hence aiding in the eradication of leftover tumor cells. These studies demonstrate encouraging outcomes, indicating that certain patients experience extended intervals without recurrence following the combined treatment of PDT and resection. Furthermore, it concludes that patients receiving higher light doses exhibit prolonged survival relative to those receiving lower doses, and that there is no associated risk for patients who undergo PDT in conjunction with postoperative RT [134, 135].

Currently, PDT is being investigated as a complementary option for the treatment of GBM. Although there are very few direct comparative studies between PDT and other treatment modalities such as CTX, RT, or surgery, research has been conducted to evaluate the efficacy of PDT in combination with these treatments. An autopsy study was conducted on GBM patients who underwent PDT and surgical resection. This study assessed the therapeutic tissue depth of PDT using sodium talaporfin and a semiconductor laser in GBM, based on autopsy findings. Three patients—one newly diagnosed and two with recurrent GBM, received intraoperative PDT along with adjuvant therapies. While no local recurrence was observed in pre-mortem imaging, autopsy findings revealed tumor recurrence within the therapeutic depth in one case. Histopathological analysis showed PDT effects extending 9–18 mm from the resection cavity, characterized by glial scarring and immune infiltration. However, viable tumor cells were detected beyond this range, highlighting the need for strategies to enhance PDT efficacy in GBM [136].

While these studies offer promising insights into the use of complementary therapies such as PDT for GBM treatment, it is essential to conduct direct comparative clinical trials to establish their relative efficacy compared to standard treatment modalities.

## Limitations and opportunities of PDT against malignant gliomas

One of the main components of PDT is the use of O_2_ from the surrounding environment, which reacts with the PS employed. Therefore, solid tumors like GBM, which exhibit low O_2_ tension, become one of the primary limitations of PDT [32]. Low O_2_ concentrations, below 1 μM, in the TME underscore the challenges and reduced efficacy of PDT, as O_2_ is essential for the therapy’s optimal performance [137]. Given that the impact of low O_2_ tension on certain tumors has been recognized, negatively influencing PDT, research has demonstrated that modulating light fluence can favorably alter tissue oxygenation, thereby improving the therapeutic response [36, 138].

**Figure 4:**
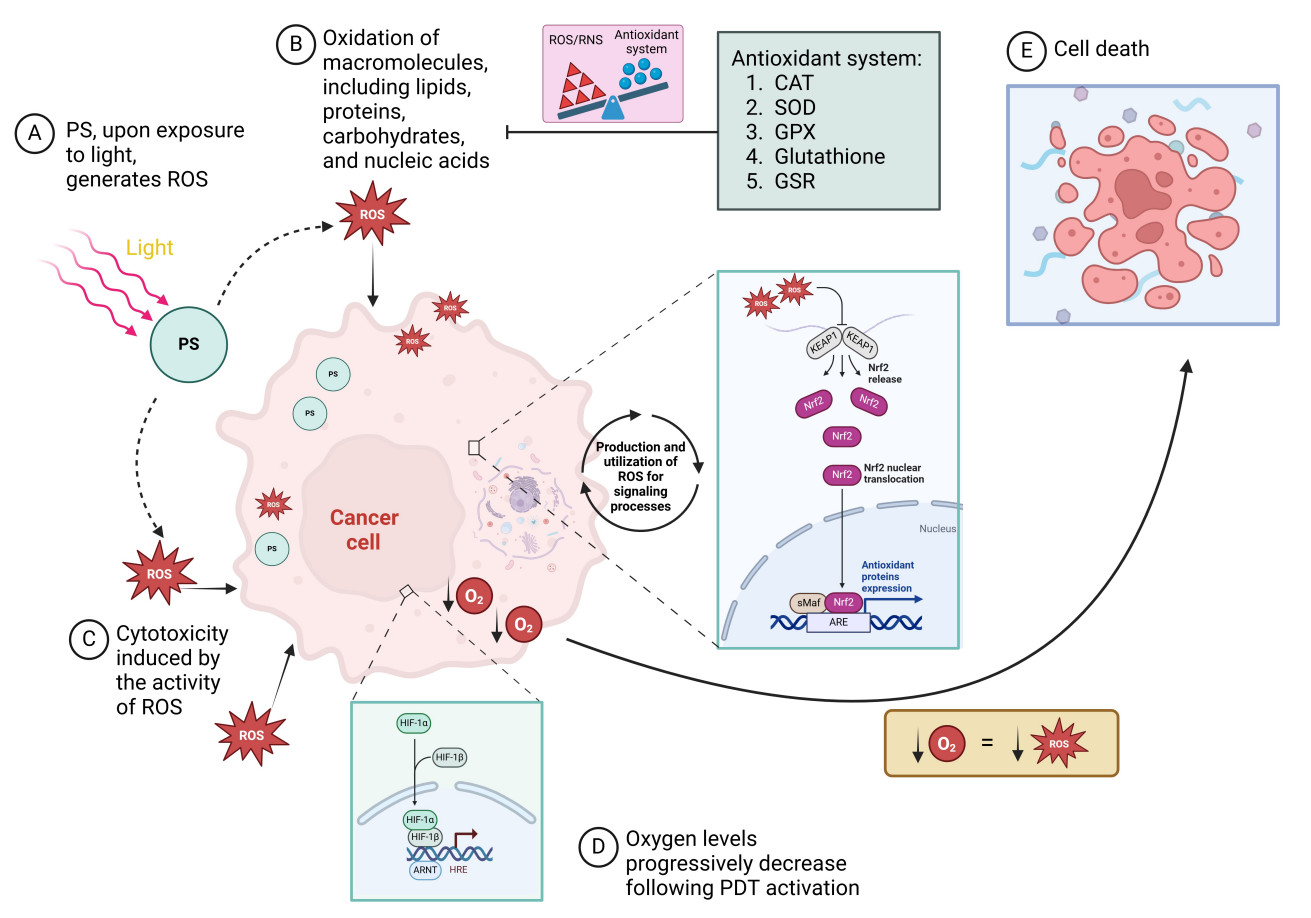
**Cellular mechanisms in response to the photodynamic effect of reactive oxygen (O_2_) species (ROS) generation and O_2_ consumption.** (**A**) Photosensitizer (PS) molecules are activated upon exposure to light, generating ROS in the presence of O_2_. (**B**) The generated ROS oxidizes essential macromolecules, such as lipids, proteins, carbohydrates, and nucleic acids, damaging cancer cell structures. (**C**) This oxidative stress leads to cytotoxicity and disrupts normal cellular function in the best of scenarios. (**D**) As O_2_ levels progressively decrease due to ROS production, hypoxic conditions activate the HIF-1 pathway, and ROS level activates the Nrf-2 pathway, further influencing cell survival and death mechanisms. (**E**) Cancer cell death occurs when the balance between ROS or reactive nitrogen species (RNS)-induced damage and the cell’s antioxidant defense systems [including catalase (CAT), superoxide dismutase (SOD), glutathione peroxidase (GPX), glutathione, and glutathione reductase (GSR)] is disrupted. The diagram illustrates the role of key cellular pathways and antioxidant mechanisms in response to photodynamic therapy (PDT)-induced oxidative stress. ARE: antioxidant response element; ARNT: aryl hydrocarbon receptor nuclear translocator; HIF: hypoxia-inducible factor; HRE: hypoxia-responsive element; KEAP1: Kelch-like ECH-Associated Protein 1; Nrf2: nuclear factor erythroid 2-related factor 2; sMaf: small musculoaponeurotic fibrosarcoma proteins. ↓: reduction/decrease. Created in BioRender. Ibarra, L. (2025) https://BioRender.com/i85f077

The regulation of redox regulatory pathways in tumor cells has been acknowledged as an effective method for their elimination [139]. The production of ROS, specifically ^1^O_2_, near the tumor site is essential for tumor eradication, as it facilitates focused and concentrated action of ^1^O_2_. However, PS will convert O_2_ into ROS with cytotoxic potential, which causes O_2_ consumption and further aggravates tumor hypoxia, resulting in decreased PDT action (Figure 4). On the other hand, the rapid growth of the tumor, O_2_ consumption exceeds its supply, potentially leading to chronic hypoxia as the distance from the tumor-associated vasculature increases [140]. Therefore, in addition to pre-existing tumor hypoxia, the efficacy of PDT will be further impaired by the O_2_ consumption involved in ROS generation [141]. Due to insufficient O_2_, hypoxia triggers a multitude of intricate intracellular signaling cascades, including the hypoxia-inducible factor (HIF) pathway. Additional pathways linked to hypoxia encompass PI3K/protein kinase B (AKT)/mammalian target of rapamycin (mTOR), mitogen-activated protein kinase (MAPK) [often referred to as the extracellular signal-regulated kinase (ERK) pathway], nuclear factor kappa-light-chain-enhancer of activated B-cells (NFκB), and nuclear factor erythroid 2-related factor 2 (Nrf2) [142]. HIF-1 expression is linked to a worse prognosis in cancer patients, as it positively modulates the expression of genes related to metabolism, metastasis, angiogenesis, and immunosuppression [143]. Conversely, HIF-1a is increased by Nrf2 in hypoxic conditions, hence enhancing GBM cell survival. Nrf2 also enhances the expression of vascular endothelial growth factor (VEGF). Nrf2 demonstrates antiapoptotic, antioxidant, anti-inflammatory, and proliferative characteristics through various regulatory mechanisms [144]. Furthermore, Nrf2 is degraded in the proteasome by the action of Kelch-like ECH-associated protein 1 (KEAP1) under normal conditions (Nrf2 is ubiquitinated by the Cul3-KEAP1 E3 ubiquitin ligase complex), however, in the presence of ROS, KEAP1 is inactivated, allowing Nrf2 to be stable, and bind to antioxidant response elements, activating their cytoprotective functions [145]. Furthermore, oxidative stress-mediated activation of the transcription factor Nrf2 induces the expression of protective antioxidant genes. Nrf2-mediated regulation of the pentose phosphate pathway (PPP) affects glucose metabolism and ROS homeostasis in cancer cells [146, 147]. Studies have shown that TMZ treatment induces Nfr2 activation, and consequently, an increase in GSH concentration in tumor cells [148]. Among the main ROS detoxification mechanisms in GBM are superoxide dismutase (SOD), catalase and GPX. Furthermore, understanding the status of these antioxidant enzymes in GBM may prove key to the development of personalized therapies targeting ROS induction [93, 149]. GSH and its related enzymes are crucial in GBM for safeguarding against free radicals, radiation, CTX, and PDT [74, 93, 150]. The importance of the GSH redox cycle in drug resistance has been analyzed and it was found that TMZ-resistant cells had lower ROS and higher antioxidant capacity, with elevated levels of GSH reductase (GSR). GSR silencing increased cell sensitivity to TMZ, while its overexpression generated resistance. Modulation of redox status by GSR influenced drug resistance, suggesting that GSR is a potential therapeutic target in GBM. Furthermore, high GSR expression was associated with shorter PFS in patients [150].

Common risks of PDT encompass cutaneous and ocular photosensitivity subsequent to the administration of the PS. Nonetheless, these effects are transient and can be alleviated by evading direct sunlight exposure. A significant risk of PDT for brain tumors is uncontrolled cerebral edema, although its precise occurrence remains undefined, as it varies according to the type of PS utilized, the delivery technique, and the intensity of light applied during treatment [121].

A study assessed the efficacy of PDT utilizing HpD as an adjunctive treatment for brain gliomas. The median survival was 76.5 months for patients with anaplastic astrocytomas and 14.3 months for those with GBM. Advanced age correlated with a reduced prognosis, although laser dosages over 230 J/cm^2^ indicated enhanced survival results. No fatalities were directly associated with PDT; nevertheless, three patients experienced cerebral edema, which was addressed with standard therapies. The occurrence of cerebral edema in these patients was about 0.04% after PDT with HpD [151]. In another study utilizing Photofrin^®^ at a dosage of 2 mg/kg, all patients exhibited good tolerance to the medication. Cerebral edema was noted in 46% of cases; however, in the majority of situations (10 cases), it was minor and did not necessitate particular intervention [126].

Modifications in lighting conditions and PS dosages are critical factors currently under investigation for each type of PS; these parameters are essential for inducing tumor cell mortality while preserving healthy tissue, mitigating complications such as cerebral edema, and preventing the emergence of treatment-resistant cells [36]. Animal experimental studies examine configurations employing surgically implanted LED lights to reliably and controllably illuminate the tumor site, enabling a metronomic application of PDT through smaller, repeated doses to enhance tumor cell destruction while reducing damage to healthy tissues [135].

## Geno-toxicological effects of PS nanomaterials

Ps nanomaterials are very effective in generating ROS when exposed to light. While ROS can be beneficial in killing cancer cells, uncontrolled ROS production can damage cellular components, including DNA. ROS can induce DNA strand breakage, base alterations, and cross-linking, potentially resulting in mutations and chromosomal abnormalities in severe cases [152]. For instance, aluminium-phthalocyanine chloride tetra sulfonate (AlPcS_4_Cl), a subclass of phthalocyanine, has been widely utilized in PDT for several tumor types, with its subcellular location determining the efficacy of PDT and the mechanism of cell death. A recent study revealed that AlPcS_4_Cl, preferentially located in the nuclei, produced DNA double-strand breaks by upregulating ataxia telangiectasia mutated (ATM), a DNA damage sensor inducing cell death [153]. Nonetheless, the possible genotoxic consequences of certain PS, especially those with a predilection for nuclear localization, are increasingly concerning and require assessment. Genotoxicity denotes the capacity of a drug to inflict damage on DNA, potentially resulting in mutations, cancer, and other detrimental health consequences [154]. In the absence of a selective strategy to target tumor cells, PS may directly interact with DNA from normal or stem cells, particularly if they concentrate within the nucleus. This contact may result in physical damage to the DNA structure or disrupt DNA replication and repair mechanisms [155]. For NPs to exhibit primary genotoxicity, they must penetrate the cytosol or nuclear membrane and directly interact with DNA and its related proteins. Multiple studies have thoroughly reported the size-dependent entry of NPs and their accumulation outside endosomes and within certain cellular compartments, ultimately reaching the nuclei [156]. Nonetheless, in vitro research failed to accurately represent in vivo behavior. The distribution of PS NPs inside the body and their potential accumulation in specific tissues, such as the liver, spleen, and kidneys, may affect their genotoxic effects. From this viewpoint, PS accumulation in tissues with considerable regenerative capacity (e.g., bone marrow) might present an increased risk. The biodistribution of NP-based PS is affected by their chemical composition, sizes, and formulation, potentially leading to accumulation in bone marrow due to their small size and prolonged circulation time, hence enhancing their propensity for extravasation into highly vascularized tissues [157]. Nevertheless, limited research addresses bone marrow exposure to PS and assesses its potential harm [158, 159]. The bone marrow microenvironment, rich in hematopoietic stem cells (HSCs) and stromal cells, can sequester PS. This is particularly concerning because HSCs are highly sensitive to oxidative stress induced by PS-generated ROS. Various cell types found in different organs and tissues may be affected by NP-based PS and could be vulnerable to genotoxicity and cytotoxicity, including epithelial cells [160] and germ cells [161]. In the particular case of brain tumors, NPs can cause oxidative stress and DNA damage in neural stem cells, impairing their ability to differentiate into neurons and glial cells, leading to neurotoxicity [162].

While PS nanomaterials are highly effective in generating ROS for targeted cancer therapy, their potential to cause genotoxic and cytotoxic damage to normal and stem cells cannot be overlooked. To ensure the safe and effective use of PS nanomaterials, further research is essential to assess their long-term genotoxic and cytotoxic effects, particularly in vulnerable tissues, and to develop strategies that enhance tumor selectivity while minimizing harm to normal and stem cells.

## PSs and their combination with other therapeutics or applications

**Figure 5:**
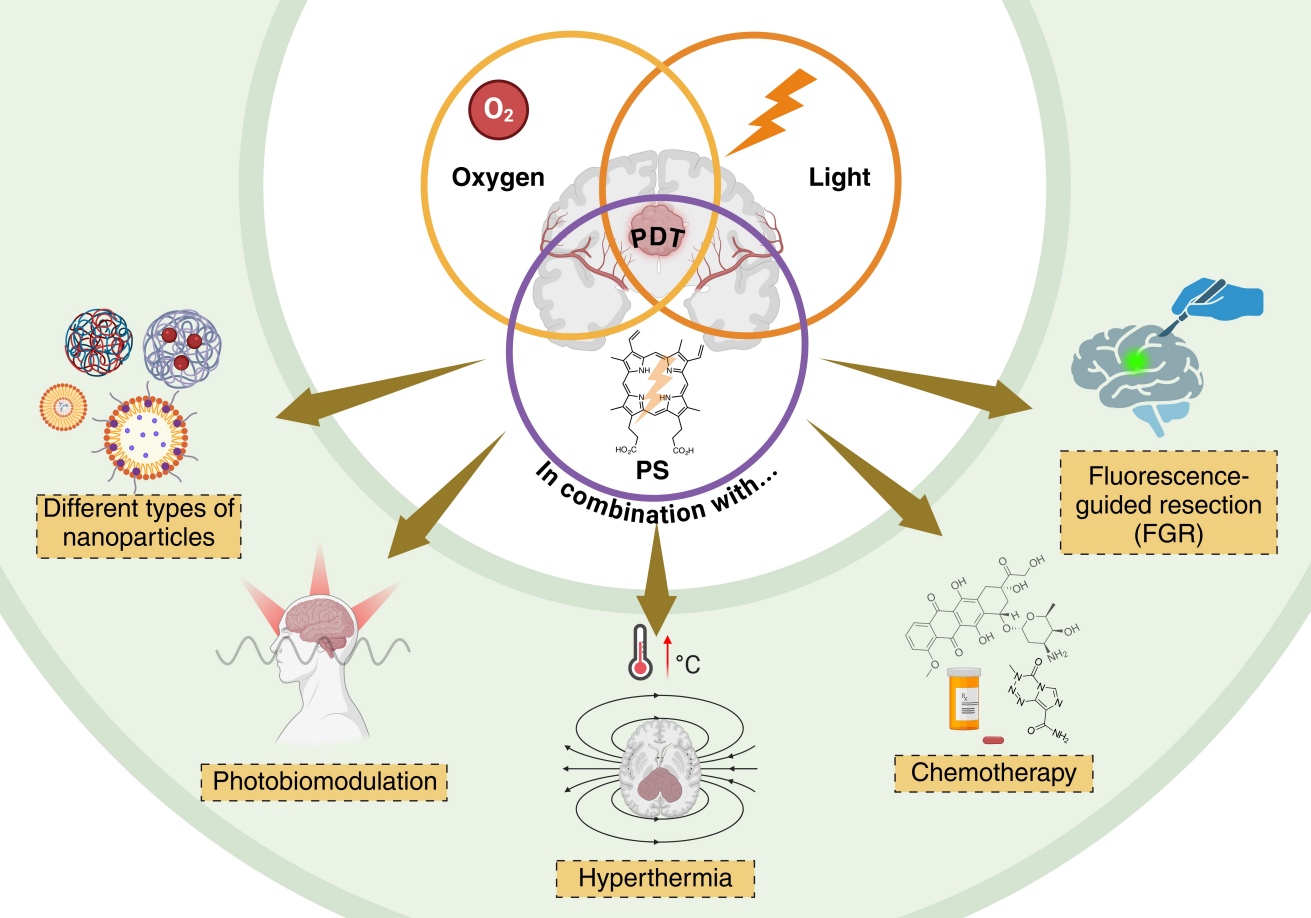
**Schematic representation of multimodal strategies for brain tumor therapy.** The central mechanism involves photodynamic therapy (PDT) targeting tumor cells with adjacent methods to enhance tumor treatment including different types of nanoparticles for targeted drug delivery, photobiomodulation to modulate cellular activity with light, hyperthermia to increase local temperature and sensitize tumor cells, chemotherapy (CTX) as a conventional treatment, and fluorescence-guided resection (FGR) for improved surgical precision. These combined approaches aim to enhance therapeutic efficacy and specificity in brain tumor treatment. Created in BioRender. Ibarra, L. (2025) https://BioRender.com/w43e824

A new direction emerges as encouraging results are obtained by combining different types of therapies, whether alternative or conventional. One therapeutic combination of interest is the application of PDT with various PSs and their integration with other therapies, whether primary or adjuvant. In recent years, synergistic effects have been observed in therapeutic combinations involving PDT, offering promising prospects for improved treatment outcomes. In this context, the following provides a brief overview of the most commonly used PS in PDT, as well as key studies on therapeutic combinations for the treatment of GBM (Figure 5).

### Combination strategies with 5-ALA and derivates

Optimistic results were observed when using FGR, a technique involving the use of a fluorescent agents that preferentially accumulate in tumor cells, aiding in the identification of areas that may not be visible to the naked eye or through conventional imaging techniques, thus increasing the likelihood of a more complete resection. FGR was combined with PDT for the treatment of GBM [121]. This study involved FGR utilizing 5-ALA in 20 patients with recurrent malignant gliomas, with laser diffusers strategically positioned within the resection cavity. PDT was administered for 60 minutes (635 nm diffuser, 200 mW/cm^2^, for 1 hour) with continuous irrigation to ensure optical clarity and a 100% O_2_ supply. MRI scans were performed at 24 hours, 14 days, and every three months post-surgery, using diffusion tensor imaging and apparent diffusion coefficient mapping. In 80% (*n* = 16) of the instances, a photodynamic therapeutic effect was noted. One patient (case 15) had surgery due to suspected GBM progression; however, histological examination indicated radiation necrosis without the presence of tumor cells. No PDT effect was seen, demonstrating a great selectivity of this treatment method for tumor cells. Subsequent MRI imaging of the group (14 days post-surgery) revealed contrast-enhanced attenuation in all patients, demonstrating a PDT impact in 80% of cases. Thus, the combination of FGR-induced PDT and 5-ALA presents an innovative and safe approach for the adjuvant treatment of local tumors, addressing the growing demand for targeted therapies. A key advantage of PDT is its ability to seamlessly integrate with existing standard treatments. Notably, 5-ALA FGR and PDT can be performed concurrently using the same drug dose. Although fluorescence may not be visible in certain tumor cells due to lower cell density, these cells remain photosensitive and can still be effectively treated with PDT. This approach enables the creation of a safety margin and facilitates the treatment of unresectable tumors located in critical areas.

Cesca et al. [74] investigated the therapeutic synergy between PDT using methyl ALA (Me-ALA), a derivative of 5-ALA, and CTX with doxorubicin (DOX), with a focus on their combined effects on redox homeostasis. They evaluated both treatments individually and in combination to assess their efficacy in inducing oxidative stress and cytotoxicity, supported by an in silico analysis that explored the roles of *PTEN* and *TP53* mutations in modulating oxidative stress. This analysis provided insights into the molecular mechanisms driving treatment responses [74]. Notably, certain mutations can enhance ROS production, leading tumor cells to generate significantly higher levels of ROS than normal cells. However, this oxidative stress is often counterbalanced by a heightened antioxidant capacity in tumor cells, developed as an adaptive response [163, 164]. GBM cell survival is heavily reliant on these antioxidant systems to mitigate the harmful effects of ROS, suggesting that inducing excessive ROS production may be an effective strategy for targeting tumor cells.

The combination of PDT and DOX demonstrated synergistic cytotoxic effects in GBM cell lines, with U87MG cells displaying the highest sensitivity to the combined treatment. The analysis of oxidative stress levels following combination therapy revealed increased oxidative stress across all cell lines, with the most pronounced effects observed in U87MG and T98G cells. These GBM cell lines, particularly those harboring mutations in tumor suppressor genes such as *TP53* and *PTEN*, often exhibit elevated baseline oxidative stress during carcinogenesis, making them highly dependent on their antioxidant systems to survive. Their findings highlight the complex interaction between pro-oxidant therapies, ROS production, and genetic factors in GBM, underscoring the importance of personalized treatment strategies targeting specific molecular pathways involved in oxidative stress response. The insufficiency of antioxidant defenses leads to oxidative stress, resulting in cell death when the harmful effects of ROS are not mitigated, suggesting that pro-oxidant therapies, such as the combination of DOX and PDT, could selectively target GBM cells, highlighting a promising pathway to improve therapeutic outcomes in GBM.

### Combination strategies with porphyrins

Verteporfin, a synthetic porphyrin and a derivative of benzoporphyrin, was assessed for its application in the treatment of GBM. Pellosi et al. [165] reported on the synergy between verteporfin-PDT and CTX with TMZ in GBM cells T98G, U87MG, and U343, using multifunctional NPs (m-NPs). The m-NPs were prepared with two types of Pluronic in various Verteporfin/TMZ ratios. Cellular uptake increased over time, reaching a plateau after four hours of incubation in all formulations, with notable improvement when biotinylated vehicles were used. Confocal microscopy analysis showed that verteporfin was distributed in the cytoplasm without reaching intracellular organelles. Given that the m-NPs did not penetrate the nucleus, the liberated TMZ was anticipated to diffuse into the nucleus, where it exerts its effects on DNA. Consequently, individual treatments did not effectively induce cell death. U87 MG and U343 cells showed greater sensitivity to TMZ compared to T98G. Furthermore, confocal microscopy studies comparing GBM cell lines with normal NIH-3T3 fibroblast cells, demonstrated that m-NPs and the encapsulated drugs were selectively taken up by tumor cells. The combinations demonstrated no toxicity in the absence of light and did not impact healthy cells; however, they exhibited a pronounced synergistic effect in cancer cells during concurrent PDT/TMZ treatment, particularly at low TMZ concentrations (0.3 mg/mL) and elevated light doses (1 J/cm^2^), as indicated by the nonlinear dose-effect curves. Consequently, the combination therapy facilitates a reduction in the dosage of TMZ while enhancing the antitumor efficacy, thereby minimizing adverse side effects.

Kang and Ko [166] conducted a study in mice xenografted with orthotopic GBM, investigating the therapeutic combination of dual selective PDT using a mitochondria-targeted PS and an optical fiber cannula. They first demonstrated the anticancer potential of albumin NPs loaded with a mitochondria-targeted PS synthesized through the conjugation of (4-carboxy-butyl)-triphenylphosphonium (TPP) and pheophorbide-a (PheoA). Previous studies have identified PheoA, a porphyrin derivative, as a key PS [167]. The TPP moiety was employed for selective mitochondrial targeting due to its cationic charge. Consequently, TPP-PheoA conjugates were developed to enhance the efficacy of PDT, with further improvements achieved by confining light delivery using an optical fiber cannula. The researchers evaluated cellular uptake in vitro and assessed the phototoxicity of NPs in U87MG GBM cells and bEnd.3 endothelial cells. In vivo biodistribution was analyzed using an in vivo imaging system (IVIS), and the photodynamic efficacy was measured through confined laser irradiation. The results were promising, indicating better uptake of NPs by U87MG tumor cells, along with greater accumulation in the brain tumor and significant tumor growth suppression following laser irradiation, both with and without the optical fiber cannula at a dose of 1 mg/kg. Notably, confined laser irradiation resulted in tumor suppression of up to 40%.

### Combination strategies with Pcs

Pcs are symmetric macrocycles formed by four isoindole units linked by nitrogen atoms. They are organic and organometallic compounds that exhibit strong and pronounced absorption in the red spectral window (> 650 nm; extinction coefficient of 105 L mol^−1^ cm^−1^), a wavelength that favors light penetration in tissue, and unlike porphyrins, they have high quantum fluorescence yields (values close to 1) [168]. Diamagnetic ions, such as Si^4+^, Zn^2+^, and Al^3+^, confer high triplet yields and long triplet state lifetimes to Pcs [169]. These PS have been studied in GBM using magnetic nanoemulsions (MNEs) loaded with citrate-coated maghemite NPs and chloroaluminum phthalocyanine (0.05 mg mL^−1^) [170] for application in HPT and PDT. The former approach involves the first exposure of tumor areas to MNPs, followed by the application of an external alternating current magnetic field, resulting in a temperature elevation of 2 to 4°C that induces cancer cell mortality through the activation of the apoptotic pathway [171]. The rationale relies on the premise that alterations in tumor temperature might impede its progression and eradicate tumor cells, hence fostering modifications that diminish transmembrane transport and destabilize their potential [87, 172, 173]. This study examined various cell lines, demonstrating that HPT treatment resulted in a 15% average reduction in cell viability across all lines, irrespective of the quantity of MNPs in the MNE. An average reduction of 52% was attained by employing two MNE formulations with varying NP quantities and administering solely the PDT light treatment. Nonetheless, the combination of HPT and PDT treatments resulted in an overall reduction of around 70%. Confocal investigations unequivocally demonstrated localization inside the cytoplasm and the active site of the drug release apparatus. Consequently, the integration of HPT and PDT therapies offers a viable strategy for brain cancer treatment.

### Combination strategies with chlorins

These PSs, relevant for PDT because of their characteristics, have a Q1 band at 650–700 nm and possess a high absorption coefficient. Consequently, Teng et al. [174] conjugated a near-infrared fluorophore [indocyanine green (ICG)] with a chlorin e6 (Ce6) on the surface of superparamagnetic iron oxide NPs (SPION) to evaluate the efficacy of PDT in vitro with GL261 cells and in vivo in a subtotal resection trial using a syngeneic flank tumor model. In vitro cellular studies demonstrated significant cytotoxicity induced by PDT using these NPs. Preclinical animal studies showed that nanoclusters could be detected through NIR imaging in both flank and intracranial GBM tumors. With these findings, the authors developed a multimodal therapeutic agent to combine PDT with optical imaging, opening the door to potential therapeutic benefits. Below are other studies combining different PSs used in PDT along with other therapies (Table 1).

**Table 1:** Overview of studies combining photodynamic therapy (PDT) with additional therapeutic strategies to overcome PDT resistance mechanisms

**Photosensitizer (PS)**	**Alternative therapy/therapeutic agent**	**Cell line or in vivo model**	**Fundamentals**	**Reference**
Aminolevulinic acid (ALA)	Photobiomodulation (PBM)	U87MG/glioma tumors grafted on the chorioallantoic membrane (CAM) of chicken embryos	Due to PBM’s enhancement of cellular metabolism, as demonstrated by increased mitochondrial ATP production, elevated oxygen (O_2_) consumption, and improved mitochondrial membrane polarization (even in hypoxic conditions), it has stimulated the investigation of PBM’s capacity to augment protoporphyrin IX (PpIX) production and improve PpIX-PDT.	[175]
5-ALA	Hypericin/PBM	U87MG	Observing that PBM induces autophagy, its application prior to hypericin was investigated, resulting in the production of plasma membrane-associated vesicles that enhanced the intracellular transport and dissolution of hypericin. This enhanced the accessibility of its physiologically active/fluorescent state for PDT, augmenting lactate dehydrogenase synthesis and improving PDT efficacy.	[39]
5-ALA	ABT-263	U251	Navitoclax (ABT-263) is an inhibitor of Bcl-2 and Bcl-xL, which are anti-apoptotic proteins, and it reinstates a pro-apoptotic phenotype. This alteration results from compromised sequestration of Bcl-2-associated X protein (BAX) and/or Bcl-2-antagonist/killer 1 (BAK) and the displacement of pro-apoptotic molecules (e.g., Noxa or BAD) from anti-apoptotic proteins within this family (e.g., Mcl-1 or Bcl-2). PDT has demonstrated a synergistic enhancement of the pro-apoptotic activity of the Bcl-2 and Bcl-xL inhibitor, ABT-263.	[176]
5-ALA	PDT combined with Acriflavine (ACF, PA)	U-251 and GL261	ACF was employed due to its selective inhibition of hypoxia-inducible factor-1 (HIF-1) activation, which is linked to oxidative stress, the mechanism of cell death induced by PDT, and the activation of several survival signaling pathways. The amalgamation of ACF with PDT diminished the expression of HIF-1a, GLUT-1, GLUT-3, and HK2, while augmenting tumor suppression, underscoring the significant function of ACF as an innovative adjuvant for PDT.	[177]
5-ALA	Biocompatible periodic mesoporous organosilica-coated Prussian blue nanoparticle (PB@PMO)	U87MG/mice	PB, a clinically utilized antidote for radioactive heavy metal toxicity, possesses the catalytic capacity to convert hydrogen peroxide (H_2_O_2_) into O_2_, hence enhancing the efficacy of PDT.	[80]
Dicysteamine-modified hypocrellin derivative (DCHB)	Multifunctional phototheranostic agent based on octadecane-modified temozolomide (TMZ-C18) for chemotherapy (CTX)	U87MG subcutaneous tumor in mice	A multifunctional phototheranostic agent is formulated using TMZ-C18 for CTX, DCHB as a natural-origin PS with a singlet O_2_ production (ΦΔ) of 0.51, and a cyclic peptide (cRGD) as a targeting unit for glioblastoma (GBM).	[178]
Hematoporphyrin monomethyl ether (HMME)	TMZ	Rat C6 glioma model using male Wistar rats	PDT markedly reduced the expression of P-glycoprotein in endothelial cells forming the blood-tumor barrier and in glioma tissues. The integration of TMZ with PDT markedly elevated TMZ levels in glioma tissues, enhanced glioma cell apoptosis, and extended the median lifespan of glioma-bearing mice.	[179]
Chlorin e6	β-Mannose	U-251	The primary justification for use of glucose in treatment is that tumor cells metabolize glucose at a higher rate than normal cells. The second objective is to enhance PS specificity by targeting tumor-associated macrophages (TAMs), which display elevated levels of the mannose receptor and promote tumorigenesis, therefore undermining therapeutic efficacy. The development of a chlorin derivative conjugated with mannose showed notable anticancer effects and enhanced PS accumulation in M2 macrophages.	[180]
5,10,15,20-tetrakis(3-hydroxyphenyl)chlorin (mTHPC)	IR-780, a photothermal agent	Murine astrocytoma (ALTS1C1)	Near-infrared radiation (NIR)-triggered PDT and photothermal therapy (PTT).	[181]
Chlorin e6	Gold nanoparticles (AuNPs)	Orthotopic GBM mice	Light-triggered PDT and PTT.	[182]

### Other combination strategies with PS NPs

Localized HPT has emerged as a promising therapeutic approach for GBM, leveraging controlled heating to enhance tumor cell sensitivity to conventional treatments. This strategy involves elevating the temperature of tumor tissues to approximately 40–45°C using various energy sources, such as radiofrequency (RF), microwaves, US, or MNPs [183–185]. HPT can induce direct cytotoxic effects by disrupting cellular homeostasis, denaturing proteins, and triggering apoptosis or necrosis in GBM cells. Additionally, it enhances the efficacy of RT and CTX by increasing drug penetration, impairing DNA repair mechanisms, and promoting immune system activation. Magnetic HPT, in particular, has shown potential in preclinical and clinical studies, utilizing functionalized MNPs that accumulate within the tumor and generate heat upon exposure to an alternating magnetic field (AMF) [183]. For instance, effective combination studies in this domain originate from the research by Beola et al. [186] which utilized lipid-based magnetic nanovectors (LMNVs) loaded with drugs to integrate HPT and CTX for the treatment of GBM. They demonstrated that exposure to AMFs facilitated magnetic HPT, which operates synergistically with the chemotherapeutic drug. Experiments on orthotopic U-87 MG-Luc2 human tumors implanted in nude mice demonstrated that glioma-targeting peptide angiopep-2 (Ang2)-TMZ-LMNV can localize and persist within the tumor following local administration, without disseminating to healthy tissue, effectively inhibiting tumor invasion and proliferation, and markedly prolonging median survival time when used in combination with AMF stimulation. Furthermore, a study conducted by Shirvalilou et al. [187] indicated that the integration of HPT with RT in GBM may enhance survival rates in GBM patients compared to RT monotherapy, especially when utilizing intratumoral injection of MNPs.

In a recent study, researchers developed a multifunctional nanodrug (MND) designed to enhance glioma treatment through a synergistic approach combining PDT, chemodynamic therapy (CDT), and CTX. The core of this nanodrug consists of up-conversion NPs (UCNPs) capable of converting 808 nm NIR light into UV light, which activates the PS NH_2_-MIL-53(Fe) incorporated as a shell component [188]. This activation initiates PDT, targeting GBM cells. Additionally, the iron ions (Fe^3+^) present in NH_2_-MIL-53(Fe) are reduced to Fe^2+^ within the TME, reacting with overexpressed H_2_O_2_ to produce HO•, thereby facilitating CDT. The system also delivers the CTX agent DOX, which is released in response to the acidic conditions characteristic of the TME, further inhibiting glioma growth. To ensure effective delivery across the BBB and targeted action, lactoferrin (LF) is conjugated to the nanodrug’s surface. This extensive therapeutic approach exhibited considerable inhibition of orthotopic gliomas in preclinical mice, underscoring the promise of MND as a sophisticated treatment mechanism for GBM.

Other researchers recently developed an MND aimed at treating GBM employing temperature-sensitive liposomes (TSLs) for the co-delivery of an aggregation-induced emission (AIE) dye, TB1, and the CTX drug paclitaxel (PTX) [189]. TB1 enables precise tumor visualization by NIR-II fluorescence imaging when exposed to NIR light. The photothermal effect generated by TB1 under NIR irradiation concurrently induces HPT, resulting in the release of PTX from the TSLs directly at the tumor site. The integrated method of PTT and CTX has shown considerable effectiveness in suppressing GBM proliferation in preclinical models.

The exploration of multimodal therapeutic approaches integrating PDT with more than two additional treatment modalities remains limited. In a study by Huang et al. [190] an MND was designed to enhance cancer therapy through the integration of CTX, PDT, and PTT. The NPs are composed of polydopamine (PDA) cores loaded with tirapazamine (TPZ), a hypoxia-activated CTX agent, and the PS ICG. Upon NIR laser irradiation, ICG generates ROS for PDT and produces localized heat for PTT. Simultaneously, PDA facilitates the release of TPZ, which becomes cytotoxic under the hypoxic conditions exacerbated by PDT and PTT. This combinatorial approach demonstrated significant tumor growth inhibition in vitro and in vivo, highlighting the potential of this MND to improve therapeutic outcomes by targeting multiple cancer cell vulnerabilities.

Additionally, the integration of sonodynamic therapy (SDT) with PDT and CTX has been explored to overcome the limitations of traditional treatments. SDT utilizes US to activate sonosensitizers (SS), producing ROS and enhancing the cytotoxic effects on cells [191, 192]. When combined with PDT and CTX agents, this approach can lead to synergistic effects, resulting in increased tumor cell death and inhibition of tumor growth. A study by Shan et al. [193] engineered a macrophage cell membrane (MCM)-cloaked MND with a ROS-responsive core composed of 4-(4,4,5,5-tetramethyl-1,3,2-dioxaborolan-2-yl) benzyl acrylate-grafted dextran (PHB-dextran). This MND co-encapsulated SS Ce6 and the bromodomain-containing protein 4 (BRD4) inhibitor JQ1. Upon ROS generation, the MND not only induced tumor cell death through SDT but also triggered ICD, activating a potent anti-tumor immune response. Concurrently, the released JQ1 inhibited tumor cell proliferation and enhanced immune activity by suppressing PD-L1 expression on tumor cells. This combined sonodynamic and immune therapy approach significantly extended the median survival time in orthotopic GL261 and *PTEN*-deficient immunosuppressive CT2A GBM mouse models, demonstrating its potential for synergistic and effective cancer treatment [193].

On the other hand, the study by Wu et al. [194] developed an MND, termed LiPTD NPs, for targeted chemo-SDT in GBM treatment. These NPs were engineered using a GSH-reactive polymer, enabling GSH depletion in the TME to enhance the efficacy of SDT. The MNDs were designed to shrink in response to neutrophil elastase in the TME, facilitating deeper tumor penetration, and were conjugated with internalizing RGD peptide (iRGD) for targeted delivery to GBM. Additionally, the MND encapsulated lexiscan enhances BBB penetration through an autocatalytic mechanism. When loaded with DOX and Ce6, NPs demonstrated efficient tumor targeting, GSH depletion, and ROS generation under US irradiation. In vivo studies showed that the combination of DOX and Ce6 delivered via LiPTD NPs significantly inhibited tumor growth and prolonged survival in GBM-bearing mice, with minimal systemic toxicity [194].

To consolidate these therapies into a cohesive treatment framework in order to reach clinical use, several critical factors must be meticulously assessed, including optimal dosages, suitable patient populations, tumor classifications, and other pertinent considerations. While combination therapies can enhance treatment efficacy through synergistic effects, they also carry the risk of increased toxicity and patient burden if not properly optimized. Careful preclinical and clinical studies are essential to determine the optimal dosages and sequencing of each therapy, particularly when these modalities are not applied simultaneously. By systematically evaluating the timing, dosage, and interaction of each therapeutic approach, researchers can identify synergistic combinations that enhance tumor targeting and reduce the risk of cumulative toxicity. This stepwise optimization is especially important in complex multimodal therapies, as it ensures that the benefits of combination treatments outweigh the potential burdens on the patient.

The integration of PDT with NP-based delivery systems presents significant challenges and limitations that must be addressed. One major concern is biocompatibility, as the materials used in MND synthesis must be carefully selected to avoid adverse immune responses or long-term toxicity in patients. While many NPs are designed to be biodegradable, their breakdown products and potential accumulation in organs, such as the liver or kidneys, raise concerns about systemic toxicity. Since these organs are the primary tissues impacted by systemically injected nanomedicines, more in-depth studies are required to evaluate the elimination pathways of these nanosystems, as well as the potential cytological and genotoxic damage to the constituent cells of these tissues due to the long-term accumulation of nanodrugs.

Additionally, the complexity of manufacturing NPs with precise size, surface properties, and drug-loading capacities adds another layer of difficulty, often requiring sophisticated techniques and quality control measures to ensure reproducibility and scalability. This challenge becomes even greater in the development of biomimetic NPs, as scaling up their production requires the procurement of biological materials and their characterization to ensure uniformity and consistency within the nanosystems. The complexity of integrating biological components, such as cell membranes or proteins, into NPs demands rigorous quality control and standardization processes to maintain functionality and reproducibility. Additionally, the variability inherent in biological materials poses significant hurdles for large-scale manufacturing, necessitating advanced techniques and protocols to achieve the desired therapeutic efficacy and safety profiles.

Another challenge lies in the TME, which can hinder the effectiveness of PDT. For instance, the hypoxic conditions commonly found in GBM tumors can limit the production of ROS, a key mechanism of PDT. Furthermore, the high levels of GSH in the TME can scavenge ROS, reducing the therapeutic efficacy of PDT. While the GSH-depleting properties of certain NPs, such as LiPTD NPs, offer a potential solution, this approach must be carefully balanced to avoid disrupting normal cellular redox homeostasis, which could lead to unintended toxicity in healthy tissues.

Moreover, the penetration of light in PDT is inherently limited by tissue depth, making it less effective for deep-seated or large tumors. Although SDT or NIR light irradiation have been explored as alternatives to overcome this limitation, the energy attenuation caused by the skull and surrounding tissues remains a significant barrier, particularly in brain tumors.

Finally, the heterogeneity of GBM poses a significant challenge, as tumors often exhibit varying degrees of resistance to therapy due to genetic and molecular diversity even into a single patient. This variability complicates the development of a one-size-fits-all treatment approach, necessitating personalized strategies that account for individual tumor characteristics. Despite these challenges, the potential of NP-based combination therapies offers a promising avenue for improving GBM treatment.

## Conclusions

Nanotechnology-based delivery systems hold immense promise for revolutionizing cancer therapy, particularly for highly aggressive and treatment-resistant malignancies like GBM. The integration of nanocarriers with PDT has already demonstrated significant advancements, including enhanced tumor targeting, reduced systemic toxicity, and multifunctional capabilities for theranostic applications. However, the road to clinical translation is fraught with challenges. Current hurdles, such as overcoming the BBB, addressing tumor-associated hypoxia, ensuring NP clearance, and reducing manufacturing complexities, must be systematically addressed. The heterogeneity of tumor vasculature and the intact BBB in certain glioma subtypes limit effective delivery of nanocarriers. Tumor-associated hypoxia, a hallmark of GBM, further reduces the efficacy of ROS-based therapies. Furthermore, insufficient clearance data on NPs from the body raises safety concerns, especially regarding potential long-term toxicity. Other barriers include the complexity and cost of large-scale NP production, the risk of off-target effects, and variability in therapeutic efficacy across different patients.

Future research should focus on designing smarter, m-NPs capable of adapting to the TME while ensuring patient safety and treatment efficacy. Innovative strategies, such as incorporating O_2_-releasing mechanisms, hypoxia-activated PSs, and BBB-penetrating delivery systems, can significantly enhance therapeutic outcomes. The integration of molecular diagnostics with advanced nanotechnology could pave the way for personalized, precision-based therapies, tailoring treatments to individual patient profiles.

As the field progresses, collaboration between multidisciplinary teams of chemists, biologists, clinicians, and engineers will be crucial to overcome current limitations and unlock the full potential of nanomaterial-based delivery systems. With continued innovation and rigorous validation, these technologies could transform the landscape of cancer therapy, offering hope for improved survival and quality of life for patients with devastating diseases like GBM.

A field to explore is the economic evaluation of its implementation in the clinic. Many PSs have been developed in recent years that deserve validation and exploration in clinical trials for approval by regulatory agencies as an alternative or complement to conventional treatments.

## Abbreviations

^1^O_2_: singlet oxygen
5-ALA: 5-aminolevulinic acid
AlPcS_4_Cl: aluminium-phthalocyanine chloride tetra sulfonate
AMF: alternating magnetic field
AO: acridine orange
BBB: blood-brain barrier
BBR: berberine
CaO_2_: calcium peroxide
CDT: chemodynamic therapy
Ce6: chlorin e6
CECs: cerebral endothelial cells
CNS: central nervous system
CPNs: conjugated polymer nanoparticles
CPs: conjugated polymers
CTX: chemotherapy
Cur: curcumin
DOX: doxorubicin
EPR: enhanced permeability and retention
EVs: extracellular vesicles
FGR: fluorescence-guided resection
FGS: fluorescence-guided surgery
GBM: glioblastoma
GPX4: glutathione peroxidase 4
GSH: glutathione
GSR: glutathione reductase
H_2_O_2_: hydrogen peroxide
HCMV: human cytomegalovirus
HCTR: healthy volunteers
HGG: high-grade glioma
HIF: hypoxia-inducible factor
HO•: hydroxyl radical
HP: hematoporphyrin
HpD: hematoporphyrin derivative
HPT: hyperthermia
HSCs: hematopoietic stem cells
HYP: Hypericin
ICD: immunogenic cell death
ICG: indocyanine green
*IDH1/2*: isocitrate dehydrogenase 1 and 2 genes
KEAP1: Kelch-like ECH-associated protein 1
KPS: Karnofsky performance status
L•: lipid radical
LH: lipid with hydrogen
LMNVs: lipid-based magnetic nanovectors
MB: methylene blue
MND: multifunctional nanodrug
MNEs: magnetic nanoemulsions
MNPs: magnetic nanoparticles
m-NPs: multifunctional nanoparticles
MRI: magnetic resonance imaging
MTD: maximum tolerated dose
mTHPC: 5,10,15,20-tetrakis(3-hydroxyphenyl)chlorin
NC: nanocomposites
NIR: near-infrared radiation
NPs: nanoparticles
Nrf2: nuclear factor erythroid 2-related factor 2
O_2_: oxygen
OS: overall survival
Pcs: phthalocyanines
PDA: polydopamine
PDT: photodynamic therapy
PFS: progression-free survival
PheoA: pheophorbide-a
pHGG: pediatric high-grade glioma
PI3K: phosphatidylinositol 3-kinase
PLGA: poly(lactic-co-glycolic acid)
PpIX: protoporphyrin IX
PSs: photosensitizers
PTT: photothermal therapy
PTX: paclitaxel
Rb: retinoblastoma
RGD: arginine-glycine-aspartic acid
rHGG: recurrent high-grade glioma
ROS: reactive oxygen species
RT: radiotherapy
S: sulfate
SDT: sonodynamic therapy
SS: sonosensitizers
TME: tumor microenvironment
TMZ: temozolomide
TPE: two-photon excitation
TPP: (4-carboxy-butyl)-triphenylphosphonium
TPZ: tirapazamine
TSLs: temperature-sensitive liposomes
TTFields: tumor-treating fields
US: ultrasound
UV: ultraviolet
XL: pyropheophorbide α 17-diethylene glycol ester
ΦΔ: singlet oxygen production

## Declarations

### Copyright

© The Author(s) 2025.
